# Sperm histone H3 lysine 4 trimethylation is altered in a genetic mouse model of transgenerational epigenetic inheritance

**DOI:** 10.1093/nar/gkaa712

**Published:** 2020-10-17

**Authors:** Ariane Lismer, Keith Siklenka, Christine Lafleur, Vanessa Dumeaux, Sarah Kimmins

**Affiliations:** Department of Pharmacology and Therapeutics, Faculty of Medicine, McGill University, Montreal, Canada; Department of Pharmacology and Therapeutics, Faculty of Medicine, McGill University, Montreal, Canada; Department of Animal Science, Faculty of Agricultural and Environmental Sciences, McGill University, Montreal, Canada; PERFORM Center and Department of Biology, Concordia University, Montreal, Canada; Department of Pharmacology and Therapeutics, Faculty of Medicine, McGill University, Montreal, Canada; Department of Animal Science, Faculty of Agricultural and Environmental Sciences, McGill University, Montreal, Canada

## Abstract

Advancing the molecular knowledge surrounding fertility and inheritance has become critical given the halving of sperm counts in the last 40 years, and the rise in complex disease which cannot be explained by genetics alone. The connection between both these trends may lie in alterations to the sperm epigenome and occur through environmental exposures. Changes to the sperm epigenome are also associated with health risks across generations such as metabolic disorders and cancer. Thus, it is imperative to identify the epigenetic modifications that escape reprogramming during spermatogenesis and embryogenesis. Here, we aimed to identify the chromatin signature(s) involved in transgenerational phenotypes in our genetic mouse model of epigenetic inheritance that overexpresses the histone demethylase KDM1A in their germ cells. We used sperm-specific chromatin immunoprecipitation followed by in depth sequencing (ChIP-seq), and computational analysis to identify whether differential enrichment of histone H3 lysine 4 trimethylation (H3K4me3), and histone H3 lysine 27 trimethylation (H3K27me3) serve as mechanisms for transgenerational epigenetic inheritance through the paternal germline. Our analysis on the sperm of KDM1A transgenic males revealed specific changes in H3K4me3 enrichment that predominantly occurred independently from bivalent H3K4me3/H3K27me3 regions. Many regions with altered H3K4me3 enrichment in sperm were identified on the paternal allele of the pre-implantation embryo. These findings suggest that sperm H3K4me3 functions in the transmission of non-genetic phenotypes transgenerationally.

## INTRODUCTION

In mice and men, both fertility and paternally transmitted effects have been associated with altered epigenetic signals in sperm including DNA methylation, non-coding RNA and histones (1,2,3,4,5,6,7,8). The phenomenon of paternal transgenerational epigenetic inheritance has been demonstrated by exposing the first generation of male mice to a toxicant or stress, and showing altered phenotypes in the two subsequent generations of unexposed offspring (9,10). While mechanistic evidence for similar effects in men and their descendants is lacking, epidemiological studies illustrate similar patterns of inheritance which are thought to be driven by epigenetic mechanisms (11). How such exposures are coded in sperm and what molecular epigenetic signatures escape epigenome reprogramming to transmit phenotypes transgenerationally remains unknown (12,13). Identification of the epigenetic signatures and the mechanisms that are transmitted from sperm to impact the health of descendants will allow for better assessment of paternal effect models especially in the context of toxicant screening, and for improved preconception advice aimed at fathers.

In *Caenorhabditis elegans*, alterations of various chromatin remodelers have been shown to induce transgenerational phenotypes through the paternal germline, highlighting the importance of histone modifications in germline and offspring development (14,15,16,17). Males bearing mutation of *spr-5*, the mammalian orthologue of LSD1/KDM1A which demethylates H3K4me1/me2, exhibited decreased fertility over multiple generations (14). Mutations of other H3K4me and H3K9me methyltransferases, demethylases, and readers, have been shown to inhibit or accelerate the cumulative transgenerational infertility phenotype in *spr-5* mutants, highlighting the complex network of histone modifiers that contribute to *C. elegans* germ cell development (16). H3K4me3-targeting chromatin modifiers have also been implicated in transgenerational phenotypes in *C. elegans*. Deficiencies in *ash-2*, *wdr-5* and *set-2* extended the lifespan of the offspring for three generations (15). Finally, male *C. elegans* carrying a mutation in the *mes-3* PRC2 subunit give rise to sterile offspring that lack H3K27me3 in their germ cells (17). These studies indicate that both active and repressive histone modifications are implicated in transgenerational epigenetic inheritance in *C. elegans*.

During mammalian spermatogenesis, a dramatic chromatin remodeling occurs whereby testis-specific histone variants are incorporated throughout meiosis and the vast majority of nucleosomes are evicted and replaced by protamines in the spermatid (18). Interestingly, sperm retain about 1% and 15% of nucleosomes in mice and men respectively (19,20,21). These retained histones are highly specific in their genomic location, being enriched at regions of high CpG density, and at genes involved in spermatogenesis, metabolism and development (6,22). Recent studies including ours indicate that histone H3 lysine 4 methylation (H3K4me) in sperm may be instructive in terms of early gene expression in the developing embryo (1,23,24,25).

**Figure 1.**
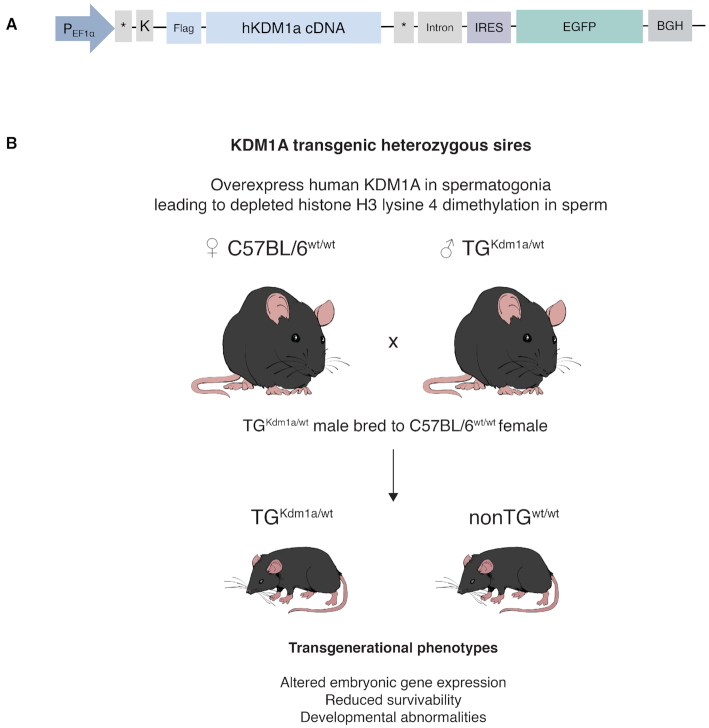
Transgenic mouse model overexpressing the histone demethylase KDM1A during spermatogenesis is a robust genetic tool to identify the chromatin marks that are associated with transgenerational epigenetic inheritance. (**A**) Construct used to generate transgenic (TG) male mice on a C57BL6/J background that overexpress human KDM1A during spermatogenesis as described in Siklenka *et al.* (1). Human KDM1A is expressed under the germ-cell specific truncated human elongation factor 1 alpha promoter (tEF1a). Kozak sequence (K), internal ribosomal entry site (IRES), enhanced green fluorescent protein (EGFP), and bovine growth hormone (BGH), are found in the polyadenylation signal. (**B**) Schematic of the transgenic mouse model. Heterozygous TG males have altered histone 3 lysine 4 dimethylation (H3K4me2) at developmental genes in sperm and give rise to transgenic (TG) or wildtype (nonTG) descendants with birth defects. Offspring subsequently sired by TG and nonTG males displayed developmental abnormalities spanning two generations.

In contrast to the vast majority of studies which have focused on either DNA methylation or non-coding RNA as mediators of epigenetic transmission, we have previously identified a role for sperm chromatin in transgenerational inheritance (1). Using a transgenic mouse model, we showed that alteration of histone methylation, specifically histone H3 lysine 4 dimethylation (H3K4me2) via the overexpression of the histone demethylase KDM1A, was associated with transgenerational phenotypes through the paternal germline (1). Transgenics (TG) that overexpressed KDM1A showed reductions of H3K4me2 at transcriptional start sites (TSS) at over 2000 genes in sperm. Loss of enrichment of H3K4me2 in sperm was linked to impaired fertility, abnormal gene expression in embryos, and offspring with severe birth defects. Hemizygous paternal transmission of the transgene generated both transgenic and wildtype-descendants, that we termed non-transgenics (nonTG). Despite not expressing the transgene, these nonTG sires gave rise to offspring with abnormal phenotypes that did not differ from those sired by transgenics. The phenotypes in nonTG descendants were termed transgenerational as they persisted in the offspring for two subsequent generations (Figure 1). Intriguingly, unlike in TG sperm, there were no significant H3K4me2 changes in nonTG sperm, purporting that other histone modifications may be driving the transgenerational phenotype observed in the nonTG offspring. Moreover, we identified no alterations in DNA methylation at CpG rich regions in TG or nonTG sperm (1). This genetic model of epigenetic inheritance based on an overexpression of KDM1A during spermatogenesis, is a robust tool to uncover epigenetic modifications that escape reprogramming and are involved in the transmission of transgenerational phenotypes (Figure 1A and B).

Here, we aimed to discern what histone modification(s) could be implicated in the transgenerational phenotypes observed in the KDM1A genetic model of epigenetic inheritance. Given that H3K4me2 is reduced in TG sperm at regions coinciding bivalent H3K4me3/H3K27me3 regions, these marks were likely candidates that may be impacted by overexpression of KDM1A during spermatogenesis. Using chromatin immunoprecipitation followed by whole genome sequencing (ChIP-seq) on C57BL/6J control wildtype (CRwt), TG, and nonTG mouse sperm, we determined whether H3K4me3 and H3K27me3 were altered in the sperm of TG and nonTG mice. Keeping in mind that the nonTG males do not express the transgene therefore generate sperm that is unexposed to the KDM1A overexpression, we hypothesized that if H3K4me3 and/or H3K27me3 were altered in both TG and nonTG sperm, this would suggest that these marks escape epigenetic reprogramming during development and are implicated in transgenerational epigenetic inheritance through the paternal germline. Here, we show that H3K4me3, but not H3K27me3, is altered in nonTG sperm at regions overlapping those altered in TG sperm, including at genes associated with the observed transgenerational phenotypes (1). This study indicates that sperm chromatin and specifically H3K4me3, escapes epigenome reprogramming to alter gene expression across generations.

## MATERIALS AND METHODS

### Breeding of KDM1A transgenic mice

KDM1A transgenic (TG) mice were generated on a C57BL/6J background as previously described (1) (Figure 1A and B). Briefly, full-length human KDM1A was overexpressed from a modified pIRES-EGFP vector (Clontech, USA; #6064-1) that contained a germ-cell specific isoform of the human EF1α promoter (26) (see Figure 1A for full construct description). Heterozygous transgenic males from the F3 generation (TG^3^) (1) were bred to C57BL/6J females to generate TG and nonTG offspring (Figure 1B). C57BL/6J control wildtype (CRwt) mice were age matched and obtained from Charles River Laboratories International (Wilmington, MA). Mice were provided with water and standard mouse chow ad libitum and housed in a 12-h light, 12-h dark cycle at 21°C. All animal procedures were approved by the Animal Care and Use Committee of McGill University, Montreal, Canada.

### Sperm isolation

Sperm isolation was previously described in Hisano *et al.* (27). Briefly, cauda epididymides were cut 4–8 times and placed in Donners medium at 37°C with gentle shaking for 1 h to allow for sperm to swim out. Note that the sperm in the cauda have undergone full histone to protamine replacement as there is no significant difference in histone fractions from sperm isolated from the cauda versus the vas deferens (28). The media containing cauda-isolated sperm was then filtered through a 20 μm strainer and washed with phosphate buffered saline (PBS) to remove somatic cells. Sperm purity and quantity was assessed via hemocytometer counts and was at minimum 99.5% pure. Recovery ranged from 5 to 8 million sperm cells per mouse.

### Sperm chromatin immunoprecipitation followed by sequencing (ChIP-Seq)

ChIP-Seq on sperm was performed as previously described (27) with some modifications for optimization of yield. For H3K4me3 ChIP-seq, a total of 12 millions sperm cells were pooled from three to four mice per genotype (C57BL/6J CRwt, TG and nonTG) with a total of three experimental replicates being sequenced. For H3K27me3 a total of 12 million sperm cells were pooled from three to four mice per genotype (C57BL/6J CRwt, TG and non-TG) with a total of two experimental replicates being sequenced. Pooled sperm was made accessible through dithiothreitol treatment. Mouse spermatozoa were lysed, washed with PBS, and aliquoted into 2 million cells per tube. Mononucleosome fragments were generated using 15 U MNase per tube (Roche, Switzerland, #10107921001). After 5 min at 37°C, MNase digestion was stopped using EDTA and chromatin was recovered by centrifugation at 17 000 × g. Chromatin was precleared with protein-A Dynabeads (Invitrogen, USA; Cat#10001D) for 1 h. Samples were immunoprecipitated overnight using 5 μg of H3K4me3 antibody (Cell Signaling Technologies, USA; #CST9751) or 5 μg of H3K27me3 antibody (Cell Signaling Technologies; USA, #CST9733) bound to 50 μl protein-A Dynabeads. The next day, bead-antibody complexes were washed and transferred to Eppendorf LoBind tubes on the last wash (Eppendorf, Germany; Cat# 0030108051). Samples were eluted twice using 125 μl of elution buffer with shaking at 400 rpm for 10 min at 65°C. Elutes were then treated with RNase A and Proteinase K. Immunoprecipitated DNA was recovered using Zymogen ChIP-Clean and Concentrator Kit (Zymo Research, USA; Cat# D5205). DNA quality and concentration were assessed using the Bioanalyzer High Sensitivity DNA kit (Agilent, USA; Cat# 5067-4626) and 3–5 ng of 147 bp mononucleosomes were size selected using Agencourt AMPure XP magnetic beads (Beckman Coulter, USA; Cat#A63880) following manufacturer's recommendations.

### Library preparation and Next Generation Sequencing

H3K4me3 and H3K27me3 libraries were prepared using Kapa BioSystems HTP Library Preparation kit (Roche, Switzerland; Cat# 07138008001). Sequencing was performed on Illumina HiSeq 2500. H3K4me3 libraries were sequenced using single-end 100 bp reads and H3K27me3 was sequenced using paired-end 100 bp reads.

### Preprocessing of sperm ChIP-Seq data

Quality of the FASTQ files were assessed with FastQC (v0.11.5, https://www.bioinformatics.babraham.ac.uk/projects/fastqc/). Reads were trimmed using Trimmomatic (v0.32) (29) with the parameters TRAILING = 20, and MINLEN = L, where L = (read length × 0.80). Reads were aligned to the mouse reference genome (UCSC version mm10, December 2011) using BWA (v0.7.15) (30) with the settings MEM -MP -t 10 -v 2 -c 100 for paired-end H3K27me3 datasets, or using Bowtie (v1.1.2) (31) with the settings -t -v 3 -m 100 for single-end H3K4me3 datasets. These parameters were implemented to reduce reads with multiple alignments (32). Samtools (v1.3) (33) was used to filter out unaligned reads, sort the alignment file and convert alignment files to BAM files. Read duplicates were marked using PicardTools (v2.4.1; Broad Institute, https://broadinstitute.github.io/picard/), but were not discarded. Alignment statistics generated with the R package ChIPQC (34) can be found in Supplementary Table S1. Our sperm-specific ChIP-seq produced robust coverage of H3K4me3 and H3K27me3 genome-wide except for one TG H3K27me3 sample (TG_A) which was excluded from downstream analyses (Supplementary Figure S1A and B).

### Genome-wide identification of sperm regions enriched for H3K4me3 and H3K27me3

**Figure 2.**
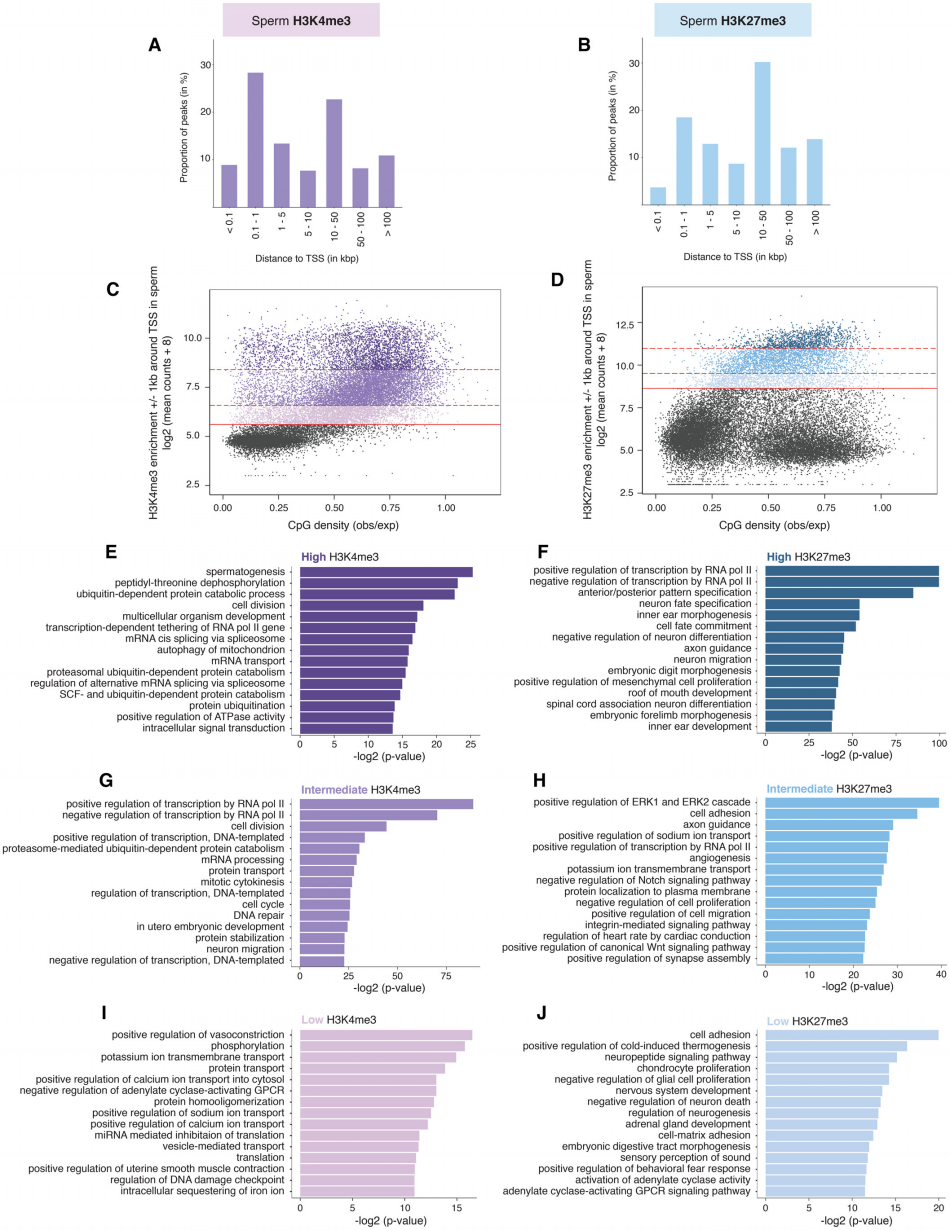
Sperm H3K4me3 and H3K27me3 are differentially distributed across the genome and are associated with different functional roles. (**A**) Proportion of H3K4me3 peaks in control wildtype (CRwt) sperm relative to the nearest transcriptional start site (TSS). (**B**) Proportion of H3K27me3 peaks in CRwt sperm relative to the nearest TSS. (**C** and **D**) Scatterplots corresponding to the log2 (mean of CRwt ChIP-Seq read counts + 8) ± 1 kb around the TSS of all known genes in the mouse genome for (**C**) sperm H3K4me3 and (**D**) sperm H3K27me3, against the CpG density of the regions (observed CpG / expected CpG). Solid red line indicates the minimum count threshold that separates enriched windows (blue) from background (black) (Supplementary Figure S3A and B; see Methods). Counts at promoter regions marked by H3K4me3 or H3K27me3 in sperm were classified into low, intermediate, and high enrichment levels based on quartile distribution cutoffs (dotted red lines separate the different quarters; see Methods). (**E**, **G**, **I**) Top significant pathways from gene ontology analysis on promoters of high (**E**), intermediate (**G**), and low (**I**) H3K4me3 enrichment quarters. (**F**, **H**, **J**) (see also Supplementary Table S3A-C). Top significant pathways from gene ontology analysis on promoters of high (**F**), intermediate (**H**), and low (**J**) H3K27me3 enrichment quarters (see also Supplementary Table S3D–F).

Regions enriched for H3K4me3 or H3K27me3 in sperm, were identified using the R/Bioconductor package csaw (v1.18.0) (35). Reads with mapping quality score above 20 were counted in 150 bp sliding windows for each library across the genome after exclusion of blacklisted regions (36). To estimate global background signal, reads were counted in 2000 bp contiguous bins for each library across the genome. We then identified regions enriched for H3K4me3 or H3K27me3 by filtering windows with background (non-specific) enrichment and by merging contiguous 150 bp windows that were remaining. All parameters were optimized independently for each mark after visual assessment of tracks using Integrative Genome Viewer (IGV) (37). Windows with a log_2_ fold change over 5 (for H3K4me3 sperm data; FigureS2A) or over 7 (for H3K27me3 sperm data; Supplementary Figure S2D) from the level of non-specific enrichment, were kept. Remaining windows less than 200 bp apart (for H3K4me3 sperm data) or 400 bp apart (for H3K27me3 sperm data) were merged. Maximum peak size for H3K4me3 and H3K27me3 sperm data was set to 7000 bp. This conferred a total of 36 418 H3K4me3 regions and 26 526 H3K27me3 regions in sperm.

### Differential enrichment analysis of H3K4me3 and H3K27me3 in TG or nonTG sperm compared to CRwt sperm

**Figure 3.**
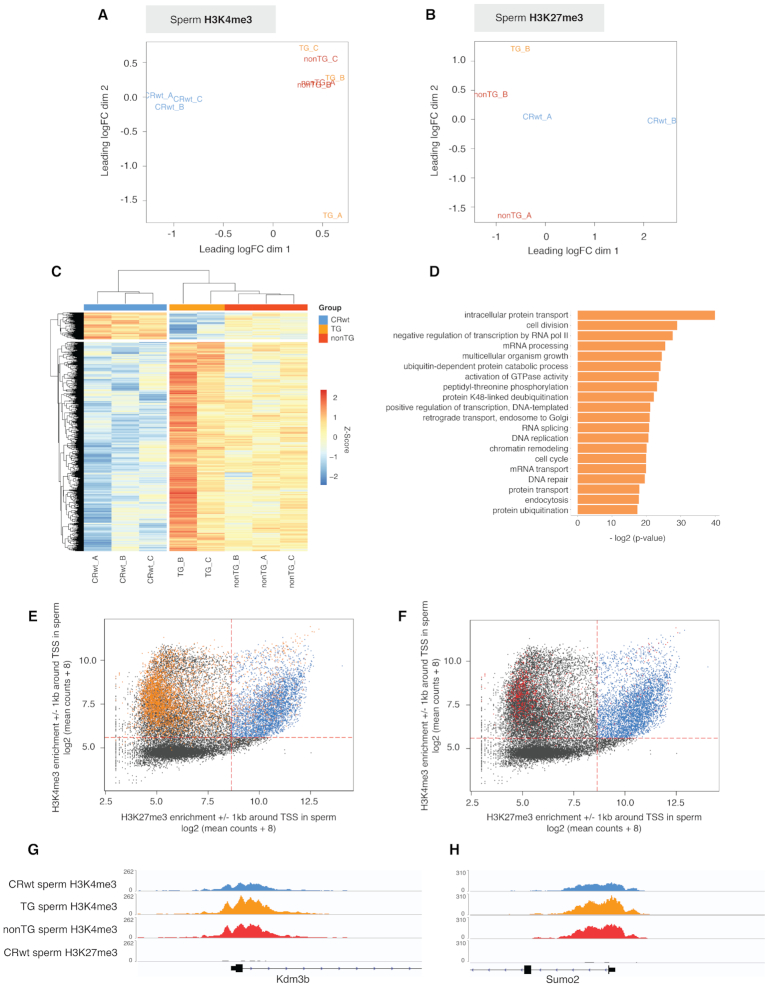
H3K4me3 is differentially enriched in TG and nonTG sperm at non-bivalent promoters. (**A**) Multidimensional scaling (MDS) plot of biological replicates for CRwt, TG, and nonTG sperm H3K4me3 normalized windows. TG_A was identified as an outlier and excluded from downstream analysis. (**B**) MDS plot of biological replicates for CRwt, TG, and nonTG sperm H3K27me3 normalized windows. TG_A failed sequencing (Supplementary Figure S1B) and could not be used for this study. (**C**) Heatmap of differentially enriched H3K4me3 (deH3K4me3) promoters in TG sperm ( = 3894 deH3K4me3 promoters, FDR < 0.2). (**D**) Top significant pathways from gene ontology analysis on promoters with deH3K4me3 in TG sperm. (**E**–**F**) Scatterplots corresponding to the log2 (mean of CRwt sperm H3K4me3 read counts + 8) ± 1 kb around the TSS of all known genes in the mouse genome against the log_2_ (mean of CRwt sperm H3K27me3 read counts + 8) ± 1 kb around the TSS, with overlay of regions with deH3K4me3 in TG sperm (orange dots, **E**) and regions with deH3K4me3 in nonTG sperm (red dots, **F**). Dashed red lines indicate the count thresholds used to separate bivalent (blue) and non-bivalent (black) chromatin in sperm (see Supplementary Figure S3A and B). Integrative Genome Viewer tracks of (**G**) the Kdm3b and (**H**) the Sumo2 promoters, that have deH3K4me3 in TG and nonTG sperm, as well as an absence of H3K27me3 in CRwt sperm.

MA-plots were used to compare the log_2_ ratio of counts per filtered windows (M) against the average abundance (A) of the window, between all samples (Supplementary Figure S2B, C, E and F). Comparisons between M values at high abundance showed a non-linear distribution for both datasets (Supplementary Figure S2B and E), suggesting a slight bias in the immunoprecipitation efficiency. These types of biases cannot be corrected by linear-scaling normalization techniques. Consequently, loess normalization was applied to the libraries under the assumption that the majority of windows are not differentially enriched (Supplementary Figure S2C and F) (38,39). Multidimensional scale (MDS) plots were generated using normalized read counts (Figure 3A and B). For the H3K4me3 dataset, TG-A was identified as an outlier on the MDS plot and was subsequently removed from further analysis. EdgeR was then used to compare H3K4me3 or H3K27me3 positive windows in TG or nonTG sperm compared to CRwt sperm (40). *P*-values were combined for all windows that overlapped ±1 kb around the TSS of known-genes using Sime's method (41) (*n* = 24 402 promoter regions; TxDb.Mmusculus.UCSC.mm10.knownGene: annotation package for TxDb objects). Benjamini–Hochberg was used for multiple testing adjustments and promoters were identified as differentially enriched for H3K4me3 or H3K27me3 using an FDR < 0.2.

### Quantification of chromatin marks and KDM1A at promoter regions

The ChIP-seq datasets used for this analysis included CRwt sperm H3K4me3 and H3K27me3, spermatocyte KDM1A (42), sperm H3K4me2 (1) and 2-cell H3K4me3 (23). Reads were counted at ±1 kb the center of the transcriptional start site of the longest transcript for all known genes (24 402 promoter regions; TxDb.Mmusculus.UCSC.mm10.knownGene: annotation package for TxDb objects). To allow for comparison of chromatin features between experiments the log_2_ of (mean counts + 8) was taken.

To identify promoters that were enriched for sperm H3K4me3, sperm H3K27me3, spermatocyte KDM1A, and sperm H3K3me2, we plotted the density distribution of the log_2_ (mean counts + 8) and identified the cutoff value between lowly and highly abundant signal based on the local minimum of the bimodal distribution (Supplementary Figure S3, Supplementary Figure S4A and B). Of note, two-cell embryo H3K4me3 density distribution did not follow a bimodal trend therefore the cutoff value between low and high abundant signal was determined by visually assessing the distribution of counts against CpG density (Supplementary Figure S4C). Promoters identified as enriched for H3K4me3 in 2-cell embryos were then inspected and confirmed in IGV.

Promoters enriched for H3K4me3 or H3K27me3 in sperm were further classified into low, intermediate, or high enrichment levels based on the quantile distribution of their densities. Low and high enrichment levels included the lowest and highest quarters of the count distribution respectively. Moderate enrichment levels comprised the two middle quarters of the count distribution.

### Gene ontology (GO) and spermatogenesis gene set enrichment analysis

GO enrichment analyses were performed using the ‘weight01’ algorithm from the R/Bioconductor topGO package (43). We also curated lists of genes expressed during the different stages of spermatogenesis (44). Each list contained the most expressed genes for the respective spermatogenesis stage based on FPKM values (top quartile, Supplementary Table S2, Supplementary Figure S5) The significance of overlap between promoters with high, medium, low levels of H3K4me3 or H3K27me3 in sperm and each list was as assessed with the hypergeometric test.

### Pre-processing of spermatocyte data and overlap with spermatocyte KDM1A regions

Raw reads for H3K4me3, H3K27me3, H3K9me2 and H3K9me3 in diplotene spermatocytes were obtained from GEO accession number GSE132446 (45). Reads were trimmed using Trimmomatic (v0.38) (29) in single-end mode (TruSeq3-SE.fa:1:30:15 TRAILING:25 MINLEN:30). Reads were aligned to the mouse reference genome (UCSC version mm10, December 2011) with Bowtie2 (v2.3.5) (46) in single-end mode using the standard settings. Samtools (v1.3) (33) was used to filter out unaligned reads, sort the alignment file and convert alignment files to BAM files. Regions enriched for KDM1A in spermatocytes were identified using the R/Bioconductor package csaw (v.1.18.0) (35) as described above. Windows with a log_2_ fold change over 6 from the level of non-specific enrichment were kept. Remaining windows <100 bp apart were merged. Maximum peak size was set to 2000 bp. This conferred a total of 31 321 KDM1A enrichment sites in spermatocytes (Supplementary Figure S6A–E).

### Pre-processing of 2-cell embryo data (23)

Raw reads for H3K4me3 and H3K27me3 ChIP-Seq in two-cell embryos obtained from GEO accession number GSE73952 (23). Reads were trimmed using Trimmomatic (v0.38) (29) in paired-end mode (TruSeq3-PE.fa:1:30:15 TRAILING:25 MINLEN:30). Reads were aligned to the mouse reference genome (UCSC version mm10, December 2011) with Bowtie2 (v2.3.5) (46) in paired-end mode using the standard settings. Samtools (v1.3) (33) was used to filter out unaligned reads, sort the alignment file and convert alignment files to BAM files. Two-cell embryo H3K4me3 or H3K27me3 enrichment signals at non-bivalent and bivalent promoters in sperm were subsequently visualized (Supplementary Figure S7).

### Pre-processing and re-analysis of Zhang *et al.* pre-implantation embryo data

Raw reads for H3K4me3 ChIP-Seq in various stages of pre-implantation embryo development were obtained from GEO accession number GSE71434(25). SNPsplit (v0.3.2; https://www.bioinformatics.babraham.ac.uk/projects/SNPsplit/) (47) was used to generate an N-masked genome using PWK SNP information (ftp://ftp-mouse.sanger.ac.uk/REL-1505-SNPs_Indels/strain_specific_vcfs/; mm10). Reads were aligned to the N-masked reference using parameters described by Zhang *et al.* (25): Bowtie2 -p 10 -t -q -N 1 -L 25 -X 2000 –no-mixed –no-discordant. Resulting BAM files were sorted using Samtools (v1.3) (33) and split into three separate alignment files containing either total, paternal (PWK) or maternal (C57BL/6J) aligned reads using SNPsplit (v0.3.2) (47).

### Bigwig coverage tracks

DeepTools2 was used to generate Bigwig coverage tracks (48). The coverage was calculated in 10 bp bins genome-wide and normalized using reads per kilobase per Million mapped reads (RPKM). Bigwig tracks were visualized in IGV.

## RESULTS

### Genomic distribution of H3K4me3 and H3K27me3 in sperm

We hypothesized that the epigenetic marks H3K4me3 and H3K27me3 may escape epigenetic reprogramming in our transgenic mouse model that overexpresses KDM1A during spermatogenesis. H3K4me3 and H3K27me3 are co-enriched at sites of H3K4me2 depletion in transgenic sperm, and bivalent H3K4me3/H3K27me3 in sperm is found at patterning genes (22) that correspond to the developmental defects (e.g. missing limbs, skeletal abnormalities) observed transgenerationally in descendants from KDM1A transgenic males (Figure 1) (1). To address this, we profiled these marks using chromatin immunoprecipitation followed by sequencing (ChIP-Seq) on the sperm of control wildtype (CRwt), transgenic (TG) and non transgenic (nonTG) males (Figure 1). Our sperm-optimized ChIP-Seq approach produced robust coverage and reproducible libraries for H3K4me3 and H3K27me3 genome-wide (Supplementary Figure S1), yielding over 30 million mapped sequencing reads for each biological replicate (Supplementary Table S1). We identified 36 418 H3K4me3 regions and 26 526 H3K27me3 regions in sperm (see Materials and Methods).

H3K4me3 was highly enriched at promoters and at loci in proximity to the transcriptional start site (TSS), with 37.2% of H3K4me3 regions located <1 kb from the TSS (Figure 2A and Supplementary Figure S8A). A large number of H3K4me3 regions were also found in intergenic space (Figure 2A and Supplementary Figure S8A). Conversely, H3K27me3 was preferentially enriched in intergenic space, with 56.2% of regions located over 10 kb from the TSS (Figure 2B). Most intergenic regions marked by H3K27me3 overlapped CpG loci (Supplementary Figure S8B). These sperm H3K4me3 and H3K27me3 genomic distributions are in line with another study that used a slightly different experimental approach which involved solubilizing the sperm chromatin with nucleoplasmin instead of digesting it with an MNase treatment (49).

To further characterize H3K4me3 and H3K27me3 enrichments at promoters in sperm, we next examined the distributions of H3K4me3 and H3K27me3 relative to CpG density ±1 kb around the TSS (Figure 2C and D). Regions enriched in H3K4me3 or H3K27me3 at these sites were defined using the local minima cutoff value of their respective density curves (Supplementary Figure S3A and B, see Materials and Methods). Concordant with previous studies, promoters bearing H3K4me3 and H3K27me3 in sperm were predominantly found at regions of higher CpG density (Figure 2C and D). To determine whether H3K4me3 or H3K27me3 enrichment levels reflected distinct functional categories at these loci, we classified both marks into high, intermediate, or low enrichment levels (Figure 2C and D, see Materials and Methods), and performed a gene ontology analysis on the promoters belonging to each category (Figure 2E–J). Promoters with high H3K4me3 enrichment mostly included spermatogenesis pathways as well as other related biological processes for sperm maturation and function such as regulation of splicing/transcription, protein modification and mitophagy (Figure 2E, Supplementary Table S3A). In particular, promoters with high H3K4me3 levels were enriched for genes solicited during late spermatogenesis stages (preleptotene spermatocytes to elongated spermatids *P* < 0.05, Supplementary Figure S5A). Promoters with intermediate H3K4me3 enrichment were involved in cell division and embryo development (Figure 2G, Supplementary Table S3B) as well as early spermatogenesis stages (primitive spermatogonia type A to pachytene spermatocytes, *P* < 0.05, Supplementary Figure S6A). Promoters with low H3K4me3 enrichment were associated with cell transport and signaling pathways (Figure 2I, Supplementary Table S3C). The promoters with the highest H3K27me3 enrichment included mostly post-implantation developmental processes such as neuron differentiation and limb development (Figure 2F, Supplementary Table S3D). The significant pathways in the moderate and low enrichment H3K27me3 categories were implicated in basic cellular processes and various developmental pathways such as skeletal system morphogenesis (Figure 2H and J, Supplementary Table S3E and F). As opposed to H3K4me3, genes expressed during spermatogenesis were associated with promoters marked by low levels of H3K27me3 in sperm (Supplementary Figure S5B, see Materials and Methods). This analysis highlights the different functional roles between sperm H3K4me3 and H3K27me3, but also reveals that their enrichment levels in sperm contribute to distinct biological processes.

### H3K4me3 is altered in the sperm of transgenic descendants that do not express the transgene and escapes epigenetic reprogramming

To understand how KDM1A overexpression during spermatogenesis impacts H3K4me3 and H3K27me3 in sperm, and to determine whether these marks are involved in transgenerational epigenetic inheritance in our genetic model, we compared H3K4me3 or H3K27me3 enrichment from CRwt sperm to TG, and nonTG sperm (35). Loess normalization was used for each ChIP-Seq dataset to correct for non-linear biases in samples and to allow for robust comparisons across experimental groups (Supplementary Figure S2C and F, see Materials and Methods) (39,40). One H3K4me3 TG sample was identified as an outlier in the multidimensional scaling (MDS) visualization (TG_A) and was excluded from the downstream analysis (Supplementary Figure S2B). Visualization of similarities between samples based on normalized counts denoted clear separation between CRwt, TG and nonTG experimental groups for H3K4me3 (Figure 3A) but not for H3K27me3 (Figure 3B). We identified 3894 H3K4me3 promoter regions differentially enriched H3K4me3 (deH3K4me3) in TG sperm compared to CRwt sperm (FDR < 0.2; see Materials and Methods), with mostly an increase in H3K4me3 enrichment in TG sperm (Figure 3C). Conversely, we identified only one promoter with differential H3K27me3 enrichment in nonTG sperm compared to CRwt sperm (FDR < 0.2, see Materials and Methods). Gene ontology analysis on the promoters with deH3K4me3 in TG sperm included pathways involved in transcriptional or translational regulation, chromatin modification, and development (Figure 3D). Strikingly, 856 promoters with H3K4me3 in sperm were differentially enriched in nonTG sperm compared to CRwt sperm, of which 75.7% overlapped promoters with deH3K4me3 in TG sperm (Figure 3C and Supplementary Figure S6F). Taken together, these findings indicate that sperm H3K4me3, but likely not H3K27me3, is altered in our genetic mouse model overexpressing KDM1A and escapes epigenetic reprogramming.

To further explore the relationship between KDM1A, H3K4me3 and H3K27me3 in sperm, we used epigenomic profiles from Chen *et al.*, (see Materials and Methods) (45) and observed that KDM1A binding sites in spermatocytes are primarily enriched in H3K4me3 but not H3K27me3 (Supplementary Figure S6A–C). This strongly suggests that H3K4me3 is more likely to be altered by KDM1A overexpression than H3K27me3 in our genetic model. While KDM1A has been shown to demethylate H3K9me2 in prostate cells (50), in spermatocytes H3K9me2 and H3K9me3 were completely absent at regions bound by KDM1A (Supplementary Figure S6A, D and E) thus making it unlikely that overexpression of KDM1A during spermatogenesis would directly alter enrichment of these marks in our model.

### Promoters with altered H3K4me3 in TG and nonTG sperm are found at non-bivalent loci

Because H3K4me3 and H3K27me3 can co-localize at genes associated with post-implantation developmental genes (6,22,51,52), we next sought to understand the relationship between deH3K4me3 in TG and nonTG sperm and H3K27me3. H3K4me3 and H3K27me3 co-localized to 4503 promoter regions in CRwt sperm (bivalent loci denoted by blue dots in Figure 3E and F). We identified a striking lack of overlap between promoters that were bivalently marked and promoters with deH3K4me3 in TG and nonTG sperm (highlighted by orange and red dots in Figure 3E and F respectively, Supplementary Figure S6F–H). In fact, only 11.2% of TG and 18.8% nonTG deH3K4me3 were detected at bivalent loci which is a significant underrepresentation of what we would expect by chance (Fisher test – odds ratios = 0.23 and 0.56, respectively; both *P* < 1.10^−12^). Examples of promoters with deH3K4me3 in TG and nonTG sperm that were mutually exclusive from H3K27me3 enrichment in sperm included the H3K9 demethylase Kdm3b (Figure 3G) and the transcriptional regulator Sumo2 (Figure 3H). Important post-implantation developmental genes marked by H3K27me3 in sperm such as Foxa1 and Hoxd11 did not have significant H3K4me3 alterations in TG or nonTG sperm (Supplementary Figure S6G, H). This analysis implies that non-bivalent promoters escape epigenetic reprogramming in male mice overexpressing KDM1A in their germ cells.

### Promoters with deH3K4me3 are targeted by Kdm1a in spermatocytes and overlap promoters with decreased H3K4me2 in TG sperm

**Figure 4.**
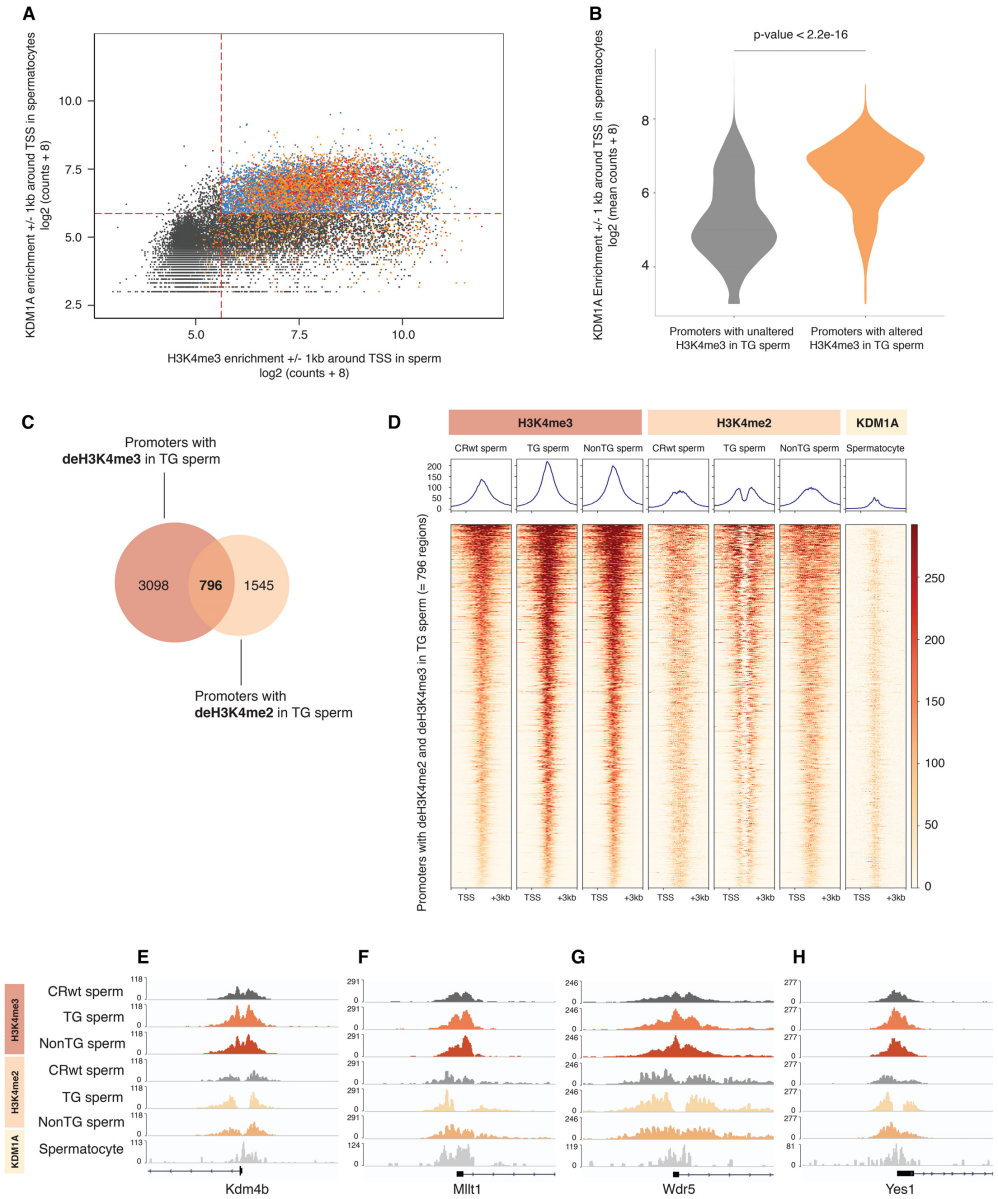
deH3K4me3 promoters in TG and nonTG sperm occur at loci bound by KDM1A in spermatocytes, and with altered deH3K4me2 in TG sperm. (**A**) Scatterplot corresponding to the log_2_ (KDM1A spermatocyte counts + 8) ± 1 kb around the TSS of all known genes in the mouse genome, with overlay of regions with deH3K4me3 in TG sperm (orange dots) and regions with deH3K4me3 in nonTG sperm (red dots). Dashed red lines indicate the count thresholds used to separate H3K4me3 regions in sperm with KDM1A binding (blue) and without KDM1A (black) in spermatocytes (see Supplementary Figure S4A and Supplementary Figure S5A, KDM1A pachytene spermatocyte dataset retrieved from Zhang *et al.*, GSE45489) (42). (**B**) Violin plots of the log2 (KDM1A spermatocyte counts + 8) ± 1 kb around the TSS of all known genes in the mouse genome relative to promoters with unaltered H3K4me3 in TG sperm (grey) or promoters with deH3K4me3 in TG sperm (orange) (Student's *t*-test, *P* < 22e–16). (**C**) Venn diagram indicating the overlap between promoters with deH3K4me3 in TG sperm and promoters with deH3K4me2 in TG sperm (1). (**D**) Heatmaps at –1 kb and +3 kb from TSS of overlapping deH3K4me2 and deH3K4me3 promoters in TG sperm (= 796 promoters) for H3K4me3 enrichment in CRwt, TG, nonTG sperm, H3K4me2 enrichment in CRwt, TG, nonTG sperm, and KDM1A enrichment in pachytene spermatocytes. (E–H) Integrative Genome Viewer tracks of (**E**) the Kdm4b, (**F**) the Mllt1, (**G**) the Wdr5 and (**H**) the Yes1 promoters, that have deH3K4me3 in TG and nonTG sperm, deH3K4me2 in TG sperm and KDM1A binding in spermatocytes.

Recent evaluations of sperm chromatin revealed that H3K4me3 and H3K4me2 are abundant in mouse sperm, with many of the same regions enriched for both modifications (1,22,51). We subsequently aimed to determine how regions with deH3K4me3 were associated with KDM1A binding in spermatocytes, and with KDM1A’s primary target H3K4me2. Intriguingly, we found that promoter regions with deH3K4me3 in TG sperm were significantly enriched at regions of KDM1A binding (Figure 4A and B). Most promoters with deH3K4me3 in TG sperm (80.5%) and deH3K4me3 in nonTG sperm (75.6%) co-localized to regions of KDM1A binding (Figure 4A). Because H3K4me3 is not a direct target of KDM1A but H3K4me2 is, we sought to elucidate whether regions with deH3K4me3 in TG sperm occurred at regions bearing H3K4me2. We found that 99.7% of regions with deH3K4me3 in TG sperm were also enriched for H3K4me2 (Supplementary Figure S4B). Moreover, 34% of promoters with deH3K4me2 in TG sperm from our previous study (1) overlapped promoters with deH3K4me3 in TG sperm from this study (Figure 4C and D). Regions with altered H3K4me2/me3 in TG sperm and strong KDM1A binding included the H3K9 demethylase Kdm4b (Figure 4E), the super elongation complex subunit Mllt1 (Figure 4F), the Wdr5 gene which associates with H3K4me and promotes its methylation (Figure 4G) (53), and the tyrosine kinase Yes1 (Figure 4H). Antibody cross-reactivity between H3K4me2 and H3K4me3 cannot be ruled out. However, the contrasting decrease in H3K4me2 due the KDM1A demethylase activity, and increase in H3K4me3 in TG sperm indicates that the anti-H3K4me3 antibody recognizes its target with a high degree of specificity. As KDM1A does not directly exert its demethylase function on H3K4me3, our results suggest that the observed H3K4me3 gains in TG sperm could be due to an imbalance between KDM1A and histone methyltransferase activity associated with H3K4me2 losses.

### H3K4me3 but not H3K27me3 in sperm promoters escape reprogramming in the embryo

**Figure 5.**
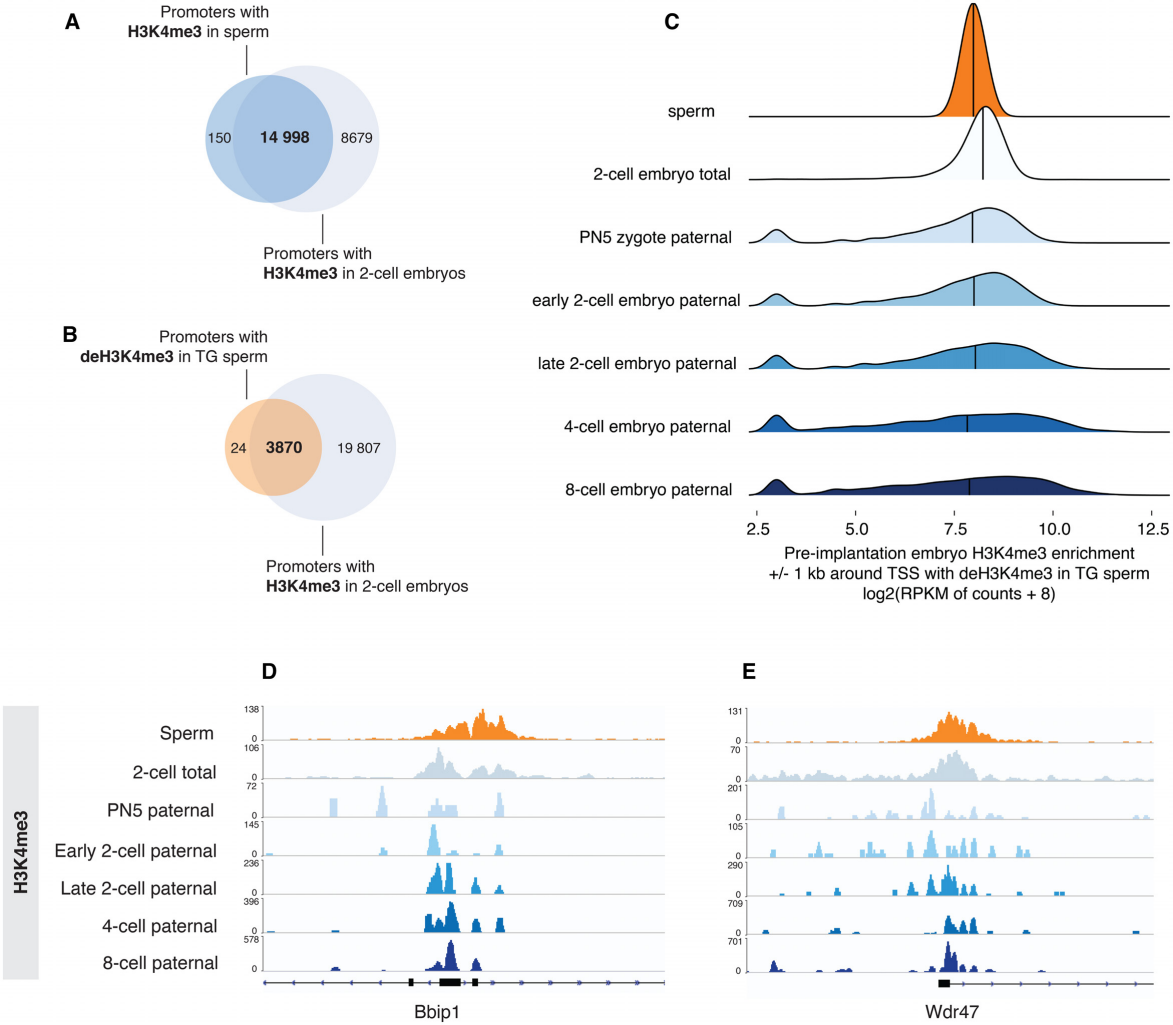
deH3K4me3 promoters in TG sperm retain H3K4me3 enrichment in the pre-implantation embryo. (**A**) Venn diagram indicating overlap between promoters with H3K4me3 in sperm and promoters with H3K4me3 in the two-cell embryo (two-cell embryo dataset retrieved from Liu *et al.* GSE73952) (23). (**B**) Venn diagram indicating overlap between promoters with deH3K4me3 in TG sperm and promoters with H3K4me3 in the two-cell embryo. (**C**) Joyplots representing H3K4me3 enrichment (RPKM + 8) in the two-cell embryo, and on the paternal allele of the pronuclear stage 5 (PN5), early two-cell, late two-cell, four-cell, eight-cell pre-implantation embryos, ±1 kb TSS with deH3K4me3 in TG sperm (two-cell embryo dataset from Liu *et al.* GSE73952, paternal allele-specific pre-implantation embryo dataset from Zhang *et al.* GSE71434. See methods for description of re-analysis of Zhang *et al.*) (25). Integrative Genome Viewer tracks of (**D**) the Bbip1 and (**E**) the Wdr47 promoters that have deH3K4me3 in TG sperm, H3K4me3 enrichment in total two-cell embryo, as well as H3K4me3 enrichment on the paternal allele of PN5 to 8-cell embryo.

To further understand whether H3K4me3 and/or H3K27me3 in sperm chromatin escape reprogramming in the embryo, we used H3K4me3 and H3K27me3 2-cell embryo data to assess how these marks at sperm promoters are enriched in the pre-implantation embryo (Supplementary Figure S7) (23). Almost all promoters enriched for H3K4me3 in sperm were also marked by H3K4me3 in two-cell embryos, suggesting a near complete retention of sperm H3K4me3 after fertilization (99%, Figure 5A and Supplementary Figure S4C). In line with this, 99.4% of promoters with deH3K4me3 in TG sperm were enriched for H3K4me3 in the two-cell embryo (Figure 5B–E). While promoters bearing H3K4me3 at non-bivalent and bivalent loci in sperm closely resembled the H3K4me3 profile in two-cell embryos (Supplementary Figure S7A–D), sperm H3K27me3 at bivalent promoters was completely absent (Supplementary Figure S7E–H). In fact, we observed a pronounced decrease in two-cell embryo H3K27me3 at promoters enriched for H3K27me3 in sperm (Supplementary Figure S7E–H). This indicates that promoter H3K27me3 in sperm is for the most part removed in the earliest stages of pre-implantation embryo development and suggests that reprogramming of H3K27me3 but not H3K4me3 takes place at regions marked by both H3K4me3 and H3K27me3 in sperm.

To investigate H3K4me3 enrichment in an earlier stage of embryogenesis prior to cell cleavage (pro-nuclear stage 5 zygote, PN5 zygote), we re-analyzed a pre-implantation embryo dataset wherein embryos were generated by breeding two divergent mouse strains, allowing for the bioinformatics discrimination between maternal and paternal alleles (25). This analysis revealed that regions with deH3K4me3 in TG and nonTG sperm were enriched for H3K4me3 on the paternal allele in the pronuclear stage 5 zygote (Figure 5C–E). Interestingly, retention of deH3K4me3 was maintained on the paternal allele throughout pre-implantation embryo development (Figure 5C–E). Taken together, this approach shows that sperm H3K4me3 is retained after fertilization and that alterations in sperm H3K4me3 can be transmitted to the pre-implantation embryo.

## DISCUSSION

Epigenetic information carried in the sperm and oocyte undergoes dramatic remodeling in the early embryo following fertilization. Sperm and oocytes have unique epigenetic signatures that must effectively be reprogrammed to allow for the transition to a totipotent zygote, capable of giving rise to all tissue and cell types. This reprogramming involves active and replication-dependent DNA demethylation as well as remodeling of the chromatin, leading to zygotic genome activation. However, this resetting of the epigenetic content of the sperm is not complete as environmentally-induced changes to the sperm epigenetic content (DNA methylation, chromatin and non-coding RNAs) have been linked to offspring phenotypes (4,54,55,56). The field of epigenetic inheritance is burgeoning with association-based studies suggesting that the mode of paternal transmission of environmental information to the offspring occurs via the sperm epigenome (57). Nonetheless, mechanistic explanations based on molecular evidence are lacking. Here, we offer the possibility that one such molecular mechanism of paternal epigenetic inheritance occurs via sperm histone H3K4me3. In our paternal genetic model of transgenerational inheritance where human KDM1A is overexpressed in the germline, we examined whether sperm H3K4me3 and H3K27me3 escape reprogramming and can thus be implicated in the transmission of abnormal phenotypes observed in the descendants of TG and nonTG males (1). The discovery of regions with abnormal H3K4me3 in the sperm of TG descendants that were unexposed to transgenic KDM1A (nonTG males), suggests that the abnormal epigenome in part escapes embryonic reprogramming, is paternally transmitted to the zygote, and maintained throughout development and spermatogenesis.

Nonetheless, it is hard to reconcile these findings with the report of Zhang *et al.* (25), which mapped paternal chromatin in sperm to the early embryo using a mouse model that allowed them to assign reads to either the maternal and paternal chromatin in order to determine the contribution of each to the early embryo (25). Interestingly they reported that regions of H3K4me3 enrichment in sperm, but not oocyte, are largely depleted from the paternal chromatin in the zygote but reappear beginning in the two-cell embryo.

If H3K4me3 is indeed mostly erased, it is unlikely that it could mediate paternal transgenerational transmission as we suggest based on our findings in this study. Upon close examination of their data and its analysis, we identified several contributing factors that led to this interpretation. We disagree with their approach for normalizing the paternal reads to the maternal ones using the equation: [maternal reads + paternal reads] / [maternal reads + paternal reads] as it does not account for the imbalance in histones found on the paternal versus maternal alleles in the zygote. In mouse sperm only 1% of histones are present as the vast majority are replaced with sperm-specific protamines (6,51). In contrast, the oocyte contains the full complement of histones with a unique broad distribution (25). An analysis without this consideration would result in an artificially decreased level of H3K4me3 on the paternal allele of the embryo. Indeed, when we re-analyzed these datasets without using a calculation for normalization to the maternal reads, we found a high degree of overlap between H3K4me3 on the paternal allele of the PN5 embryo and the closely resembling H3K4me3 regions in sperm (Figure 5C–E). As oocyte H3K4me3 is mostly found in non-canonical/intergenic space (24,25), the retention of sperm H3K4me3 at promoters in the embryo and the contrast between oocyte H3K4me3 profiles suggest a sperm-specific inheritance of H3K4me3 after fertilization.

These opposing sperm and oocyte profiles, together with our re-analysis of the Zhang *et al.* data, supports our findings that altered enrichment of H3K4me3 in sperm can lead to the phenotypes we observe in our genetic model of transgenerational epigenetic inheritance. Lending additional support to our findings, Aoshima *et al.* report that loss of H3K4 methylation in sperm disrupts minor zygotic gene activation in the paternal pronucleus (58).

At this time, it remains unknown how H3K4me3 escapes epigenetic reprogramming in both embryonic and germline development. However, our findings are in line with a role for histones in transgenerational inheritance that has been reported in yeast, fly and worm (59). Unique so far to mammals is our identification in this transgenic model that H3K4me3, but not H3K27me3, escapes epigenetic reprogramming. In yeast and fly, H3K27me3 was identified as an epigenetic signal able to mediate non-genetic inheritance between generations (60,61,62). Here we found that H3K4me3 exclusively bypassed reprogramming at regions that were not marked by H3K27me3 in sperm. The specificity of H3K4me3 non-bivalent targeting hints that bivalent H3K4me3/H3K27me3 may be protected by its association with the polycomb repressive complex (PRC). The PRC2 complex consists of EZH1/2, EED and SUZ12, which function as repressors that are essential for gene expression during embryonic development (63,64).

The histone marks H3K9me3 and H3K27me3 have been implicated in transgenerational inheritance in *C. elegans* following exposure to the endocrine disruptor bisphenol A (65). As discussed previously, we did not find evidence that H3K27me3 contributes to transgenerational inheritance in our genetic model and demonstrated that H3K27me3 at promoters in sperm was lost in the 2-cell embryo. We also noted that H3K9me2 and H3K9me3 were not enriched at KDM1A binding sites in spermatocytes. Based on our initial characterization of the model (1) we found that epigenetic alterations in TG sperm were specific to the TSS and impacted H3K4me2 but not DNA methylation. Given the inter-relationship between DNA methylation and histone H3K9me3, their co-localization to repeat regions but not promoters (22,49), and the normal suppression of LINE elements in TG sperm, we furthermore deemed H3K9me as likely not the main actor in our model despite being a target of KDM1A in prostate cells (50). Although we cannot exclude the possibility that these marks may be implicated in epigenetic inheritance in mammals, they are likely not the predominant actor in our mouse model.

Our data demonstrates a strong overlap between deH3K4me3 in TG sperm and Kdm1a binding sites identified in spermatocytes (42), yet it is unclear what factors mediate the increase in H3K4me3. It is possible that transgenic KDM1A influences the establishment of H3K4me3 during spermatogenesis indirectly through the action of KDM1A’s binding partners and/or protein complex associations (50,66,67). For example, in addition to a primarily repressive role in transcription (68,69), KDM1A has been found to associate with the methyltransferase-containing super-complex MLL1, which is involved in H3K4me3 methylation and transcriptional activation (70). Likewise, in prostate tumour cells, direct methylation of KDM1A by the protein-methyltransferase EHMT2 facilitates an interaction between the chromatin remodeling proteins CHD-1 and KDM1A, that is necessary for androgen receptor dependent transcriptional activation (50). The dual chromodomains of CHD-1 have been shown to selectively target H3K4me3 regions that are independent of any co-enrichment for H3K27me3 (71,72). This interaction would therefore support the underrepresentation of H3K27me3 at gene promoters associated with deH3K4me3 found in this model.

Here, we provide evidence for H3K4me3 as one potential mechanism underlying transgenerational epigenetic inheritance. Other key examples of paternal intergenerational epigenetic inheritance have been linked to sperm-borne non-coding RNA, but whether they participate in the stable transmission of transgenerational epigenetic phenotypes is not known (2,3,4). The next steps for the field of epigenetic inheritance are to determine the interplay between these three epigenetic players, as well as the various chromatin modifications that are implicated in transgenerational inheritance. Our data suggests that sperm-inherited H3K4me3, but not H3K27me3, escapes reprogramming in the embryo. Understanding whether altered H3K4me3 in sperm is transmitted to the embryo and deregulates gene expression is another critical question for the field to resolve.

## DATA AVAILABILITY

Sperm H3K4me3 and H3K27me3 datasets are available on Gene Expression Omnibus under the accession number GSE145679.

## Supplementary Material

gkaa712_Supplemental_Files
