# Supplementary material for: Sperm histone H3 lysine 4 trimethylation is altered in a genetic mouse model of transgenerational epigenetic inheritance
Source: Nucleic Acids Res. 2020 Oct 17;48(20):11380–93. doi: 10.1093/nar/gkaa712 (PMC7672453; doi:10.1093/nar/gkaa712)
Supplement: gkaa712_Supplemental_Files [file gkaa712_supplemental_files.zip › merged_supplemental_Figures.pdf]

Figure S1

A

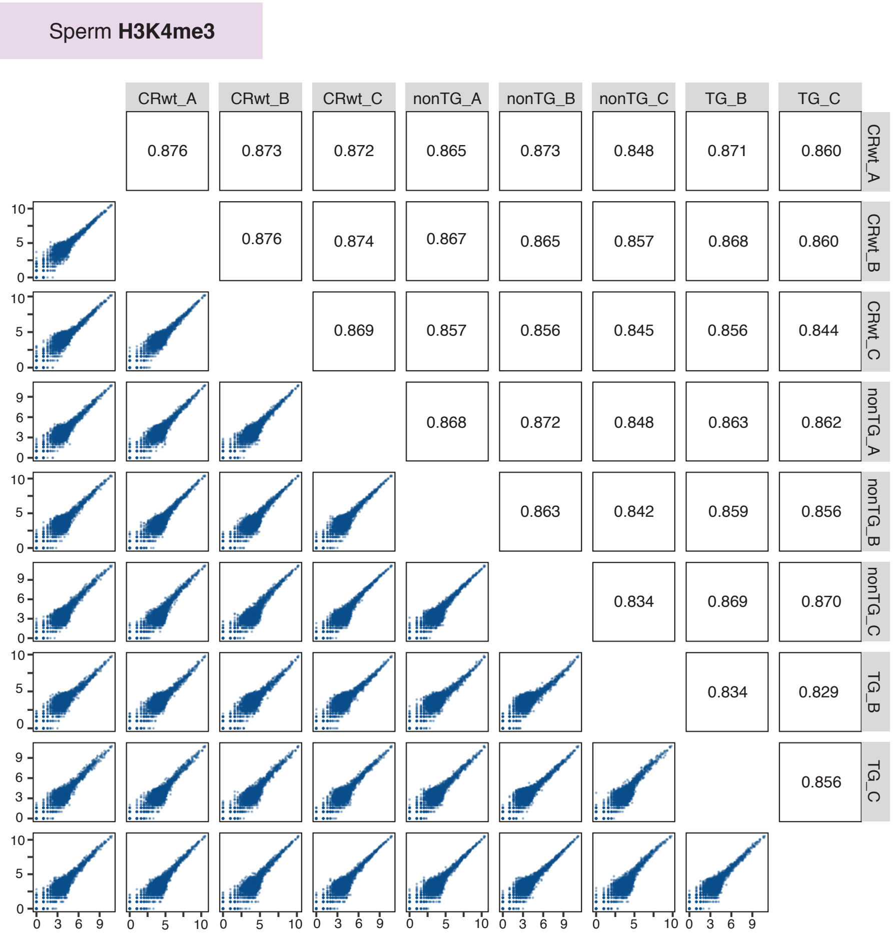

Sperm **H3K4me3**
CRwt_A
CRwt_B
CRwt_C
nonTG_A
nonTG_B
nonTG_C
TG_B
TG_C
0.876
0.873
0.872
0.865
0.873
0.848
0.871
0.860
0.876
0.874
0.867
0.865
0.857
0.868
0.860
0.869
0.857
0.856
0.845
0.856
0.844
0.868
0.872
0.848
0.863
0.862
0.863
0.842
0.859
0.856
0.834
0.869
0.870
0.834
0.829
0.856

B

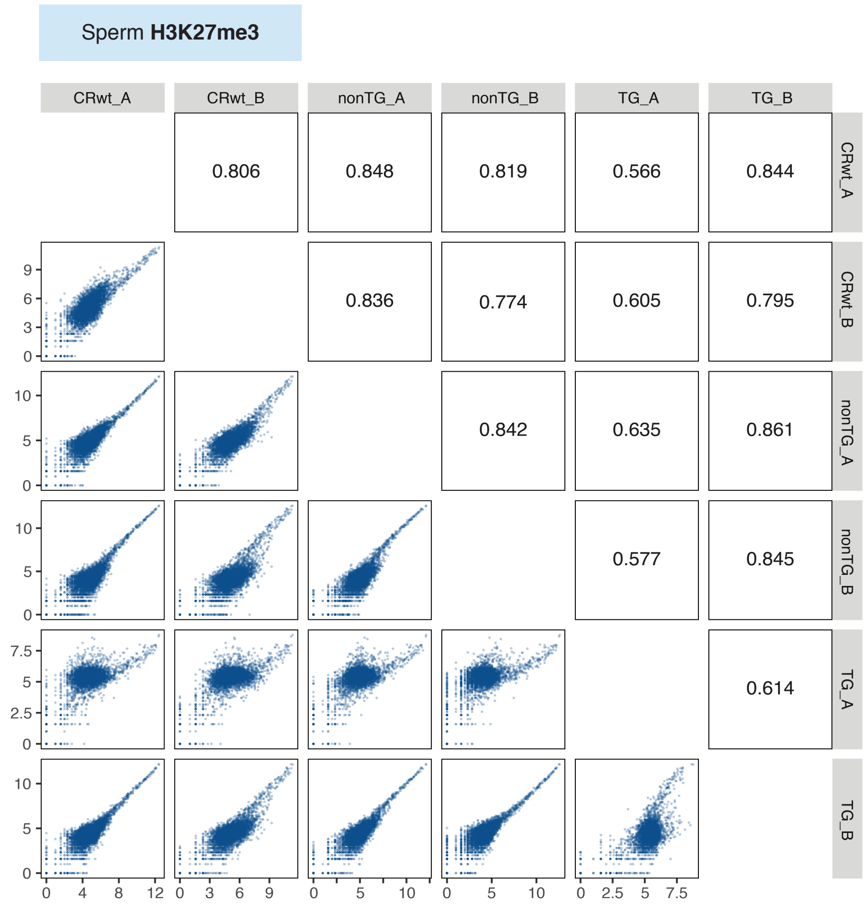

Sperm **H3K27me3**
CRwt_A
CRwt_B
nonTG_A
nonTG_B
TG_A
TG_B
0.806
0.848
0.819
0.566
0.844
0.836
0.774
0.605
0.795
0.842
0.635
0.861
0.577
0.845
0.614

Figure S2

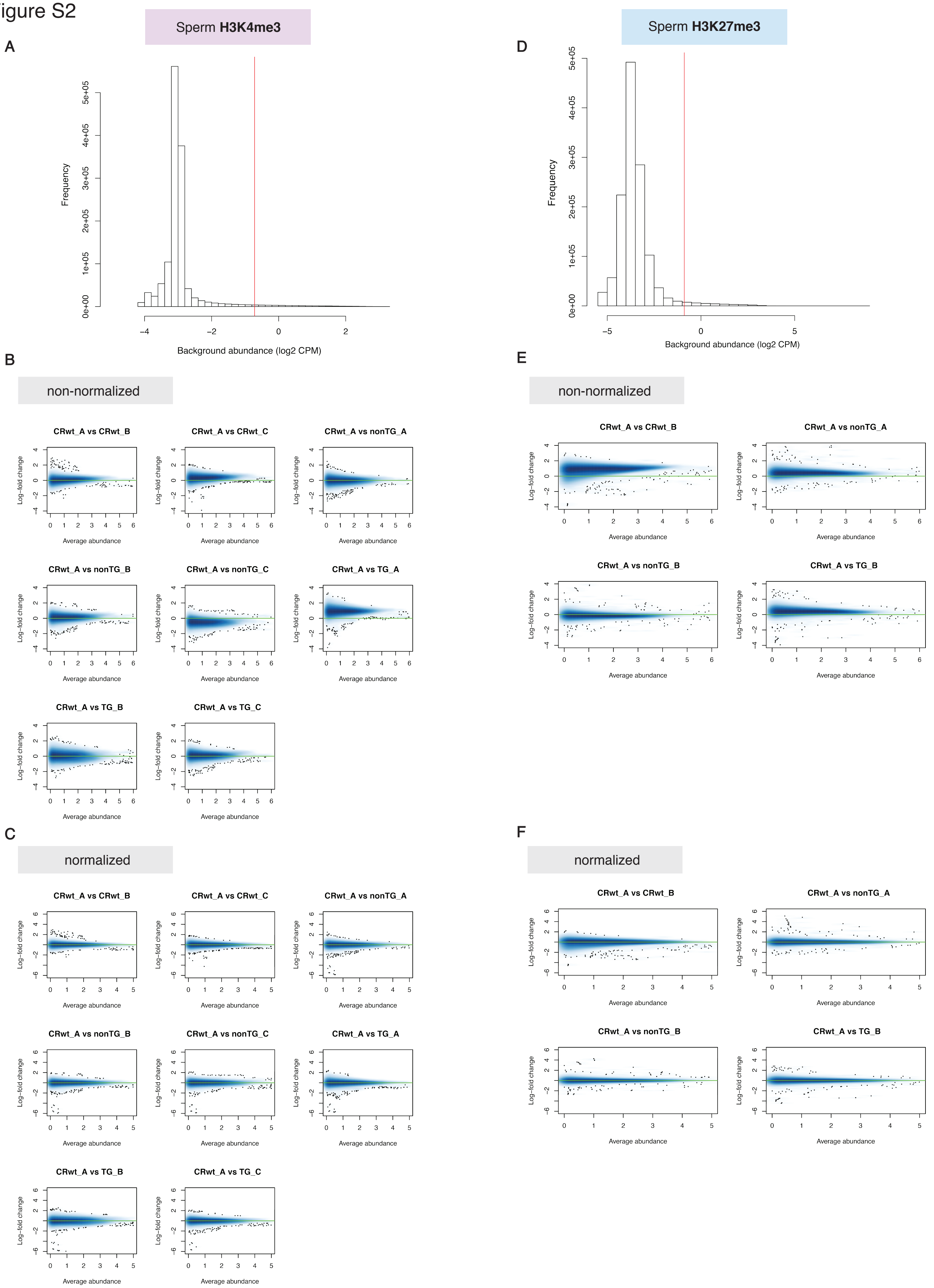

Sperm H3K4me3
A
Frequency
0e+00
1e+05
2e+05
3e+05
4e+05
5e+05
-4
-2
0
2
Background abundance (log2 CPM)
B
non-normalized
CRwt_A vs CRwt_B
CRwt_A vs CRwt_C
CRwt_A vs nonTG_A
CRwt_A vs nonTG_B
CRwt_A vs nonTG_C
CRwt_A vs TG_A
CRwt_A vs TG_B
CRwt_A vs TG_C
Log−fold change
Average abundance
C
normalized
CRwt_A vs CRwt_B
CRwt_A vs CRwt_C
CRwt_A vs nonTG_A
CRwt_A vs nonTG_B
CRwt_A vs nonTG_C
CRwt_A vs TG_A
CRwt_A vs TG_B
CRwt_A vs TG_C
Log−fold change
Average abundance
Sperm H3K27me3
D
Frequency
0e+00
1e+05
2e+05
3e+05
4e+05
5e+05
-5
0
5
Background abundance (log2 CPM)
E
non-normalized
CRwt_A vs CRwt_B
CRwt_A vs nonTG_A
CRwt_A vs nonTG_B
CRwt_A vs TG_B
Log−fold change
Average abundance
F
normalized
CRwt_A vs CRwt_B
CRwt_A vs nonTG_A
CRwt_A vs nonTG_B
CRwt_A vs TG_B
Log−fold change
Average abundance

Figure S3

A

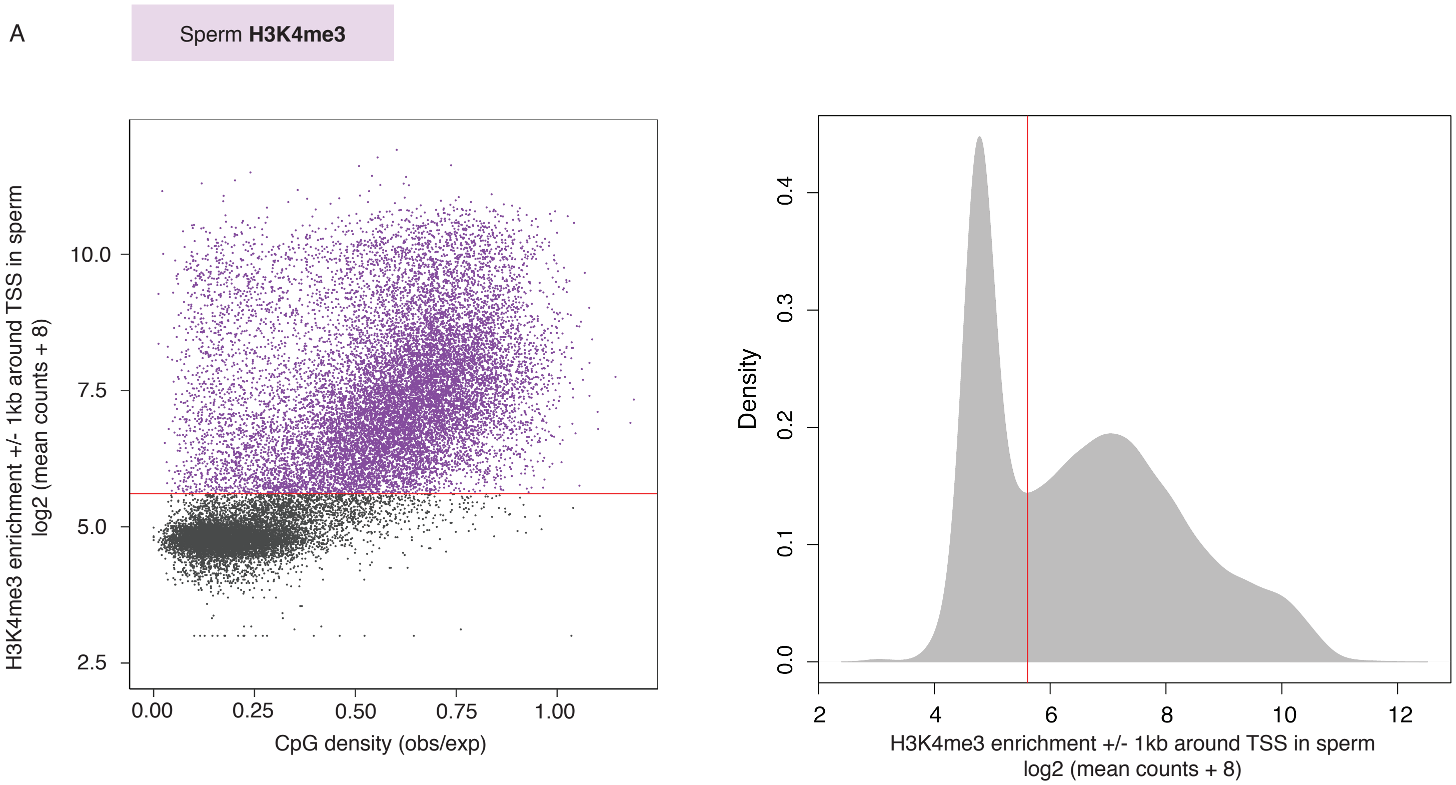
Sperm H3K4me3
H3K4me3 enrichment +/- 1kb around TSS in sperm
log2 (mean counts + 8)
10.0
7.5
5.0
2.5
0.00
0.25
0.50
0.75
1.00
CpG density (obs/exp)
Density
0.4
0.3
0.2
0.1
0.0
2
4
6
8
10
12
H3K4me3 enrichment +/- 1kb around TSS in sperm
log2 (mean counts + 8)

B

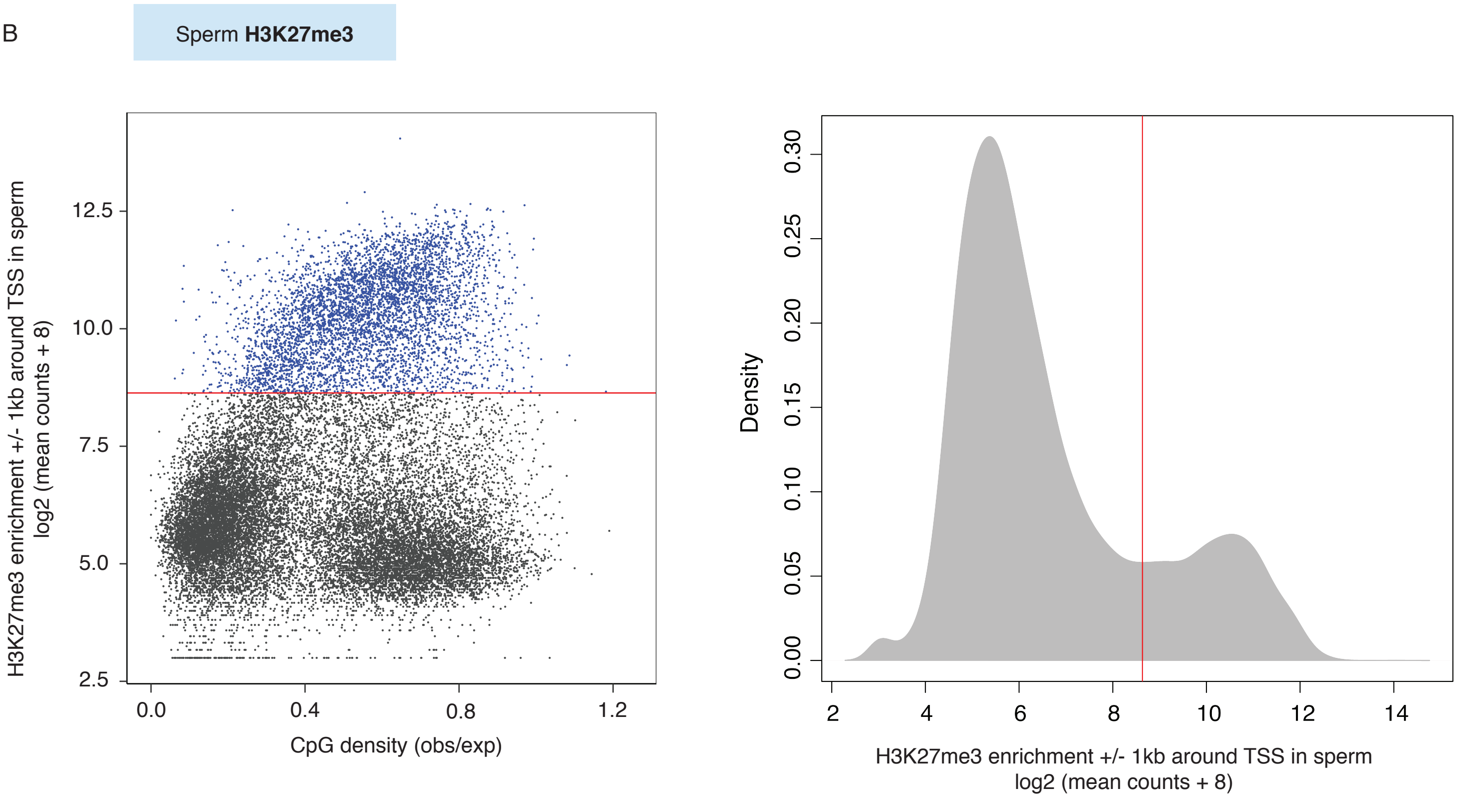
Sperm H3K27me3
H3K27me3 enrichment +/- 1kb around TSS in sperm
log2 (mean counts + 8)
12.5
10.0
7.5
5.0
2.5
0.0
0.4
0.8
1.2
CpG density (obs/exp)
Density
0.30
0.25
0.20
0.15
0.10
0.05
0.00
2
4
6
8
10
12
14
H3K27me3 enrichment +/- 1kb around TSS in sperm
log2 (mean counts + 8)

Figure S4

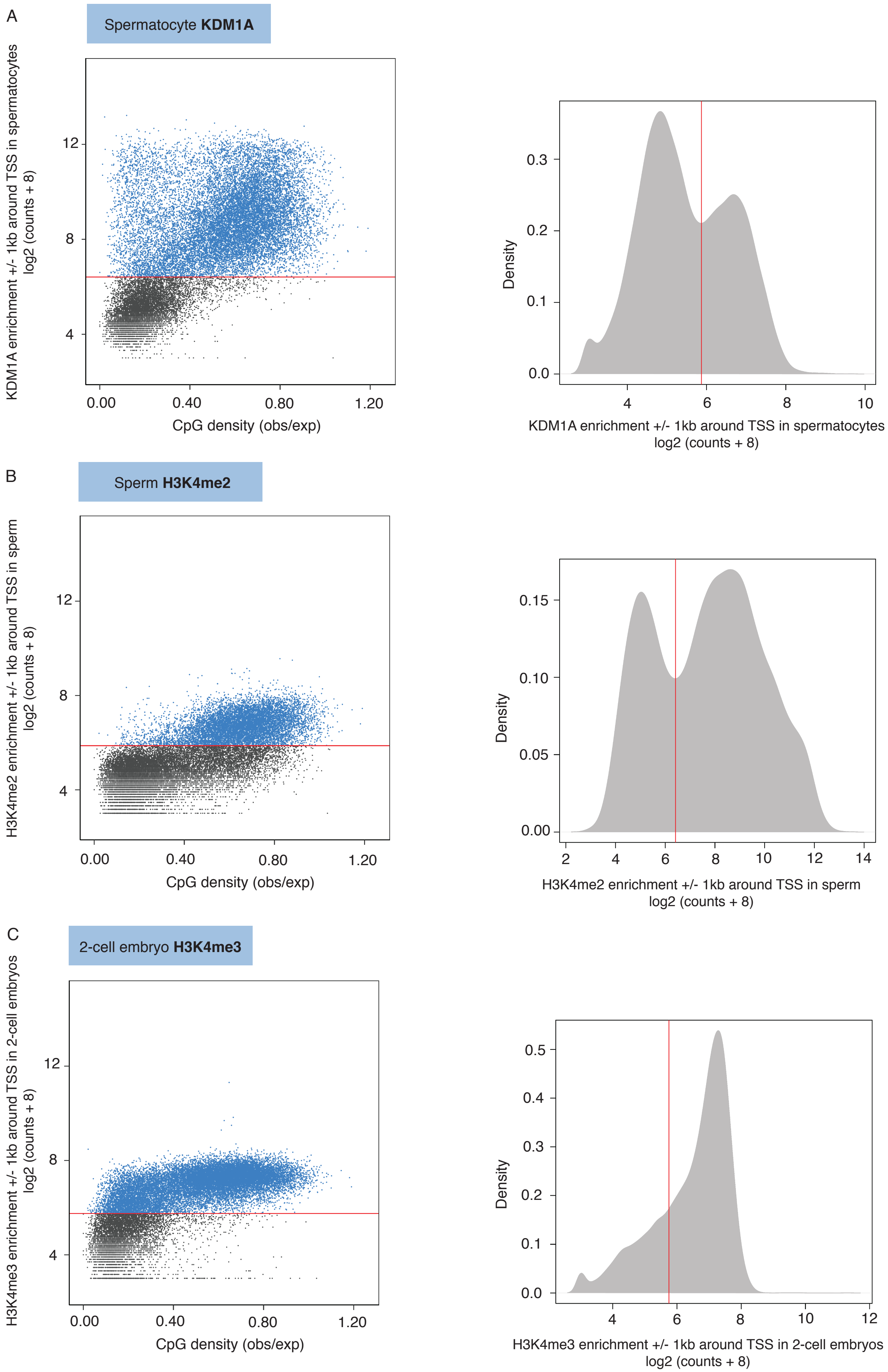

A
Spermatocyte **KDM1A**
KDM1A enrichment +/- 1kb around TSS in spermatocytes
log2 (counts + 8)
12
8
4
0.00
0.40
0.80
1.20
CpG density (obs/exp)
Density
0.3
0.2
0.1
0.0
4
6
8
10
KDM1A enrichment +/- 1kb around TSS in spermatocytes
log2 (counts + 8)
B
Sperm **H3K4me2**
H3K4me2 enrichment +/- 1kb around TSS in sperm
log2 (counts + 8)
12
8
4
0.00
0.40
0.80
1.20
CpG density (obs/exp)
Density
0.15
0.10
0.05
0.00
2
4
6
8
10
12
14
H3K4me2 enrichment +/- 1kb around TSS in sperm
log2 (counts + 8)
C
2-cell embryo **H3K4me3**
H3K4me3 enrichment +/- 1kb around TSS in 2-cell embryos
log2 (counts + 8)
12
8
4
0.00
0.40
0.80
1.20
CpG density (obs/exp)
Density
0.5
0.4
0.3
0.2
0.1
0.0
4
6
8
10
12
H3K4me3 enrichment +/- 1kb around TSS in 2-cell embryos
log2 (counts + 8)

Figure S5

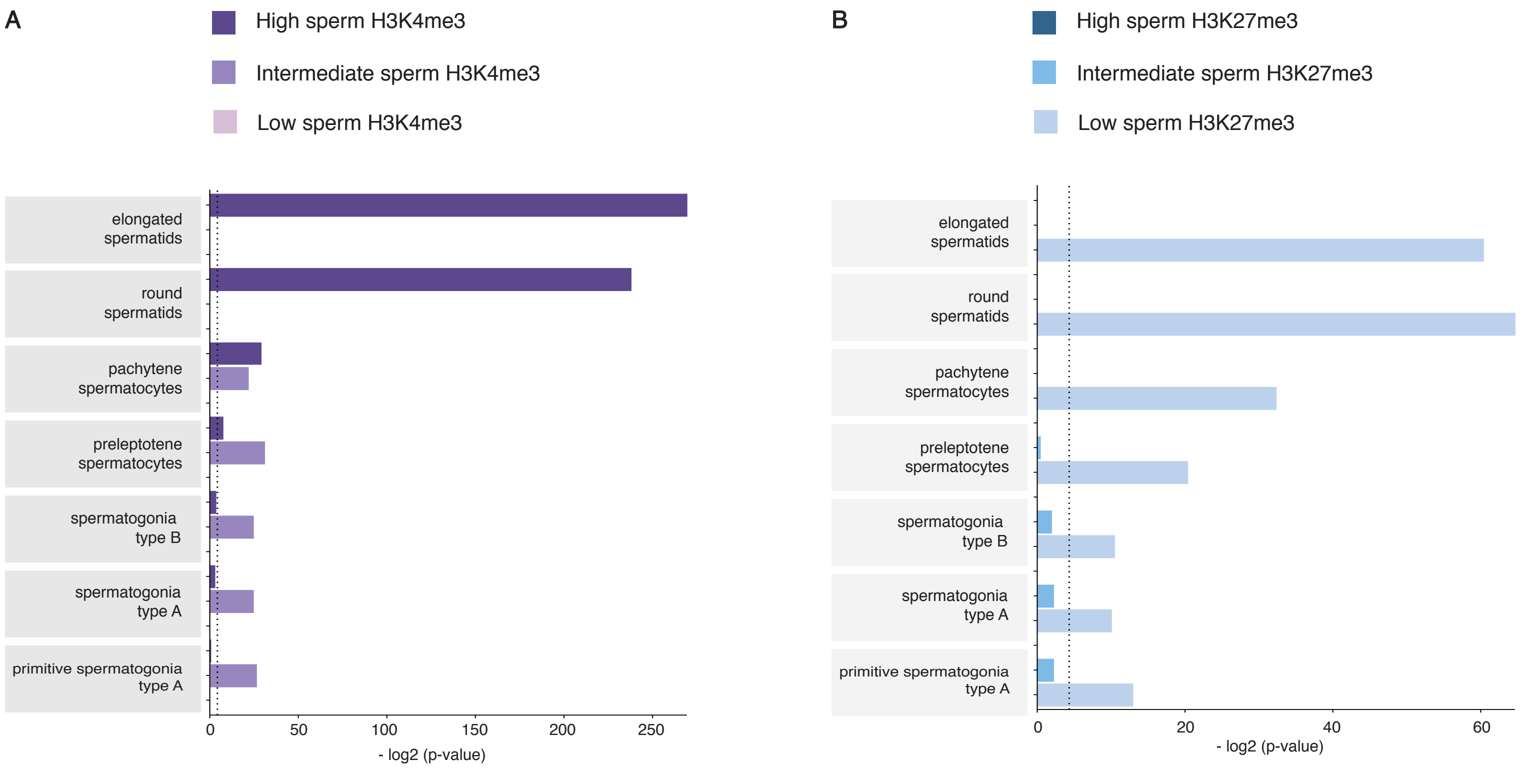
A
High sperm H3K4me3
Intermediate sperm H3K4me3
Low sperm H3K4me3
elongated spermatids
round spermatids
pachytene spermatocytes
preleptotene spermatocytes
spermatogonia type B
spermatogonia type A
primitive spermatogonia type A
0
50
100
150
200
250
- log2 (p-value)
B
High sperm H3K27me3
Intermediate sperm H3K27me3
Low sperm H3K27me3
elongated spermatids
round spermatids
pachytene spermatocytes
preleptotene spermatocytes
spermatogonia type B
spermatogonia type A
primitive spermatogonia type A
0
20
40
60
- log2 (p-value)

Figure S6

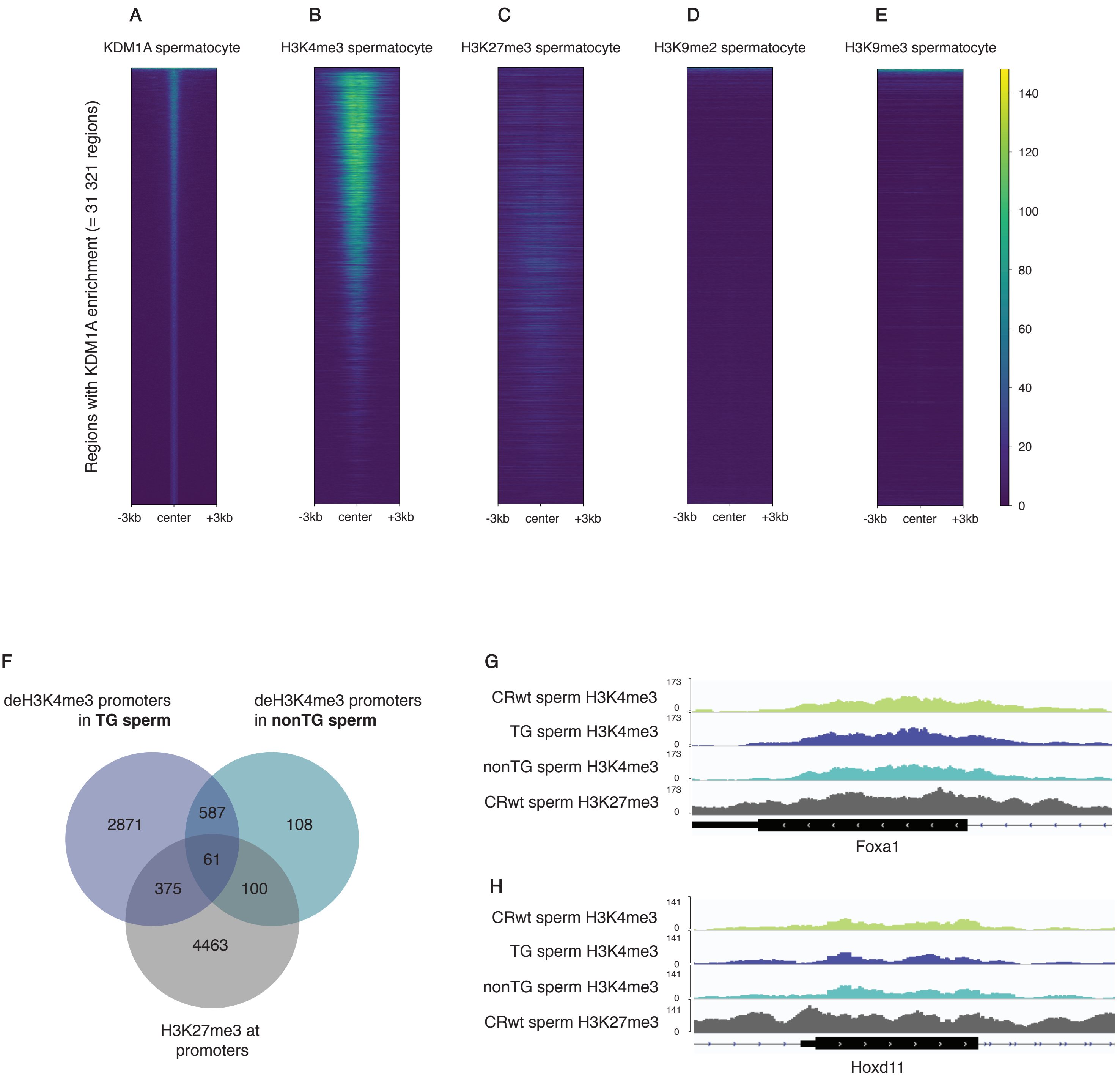
A
KDM1A spermatocyte
B
H3K4me3 spermatocyte
C
H3K27me3 spermatocyte
D
H3K9me2 spermatocyte
E
H3K9me3 spermatocyte
Regions with KDM1A enrichment (= 31 321 regions)
-3kb
center
+3kb
140
120
100
80
60
40
20
0
F
deH3K4me3 promoters in **TG sperm**
deH3K4me3 promoters in **nonTG sperm**
2871
587
108
61
375
100
4463
H3K27me3 at promoters
G
CRwt sperm H3K4me3
TG sperm H3K4me3
nonTG sperm H3K4me3
CRwt sperm H3K27me3
173
0
Foxa1
H
CRwt sperm H3K4me3
TG sperm H3K4me3
nonTG sperm H3K4me3
CRwt sperm H3K27me3
141
0
Hoxd11

Figure S7

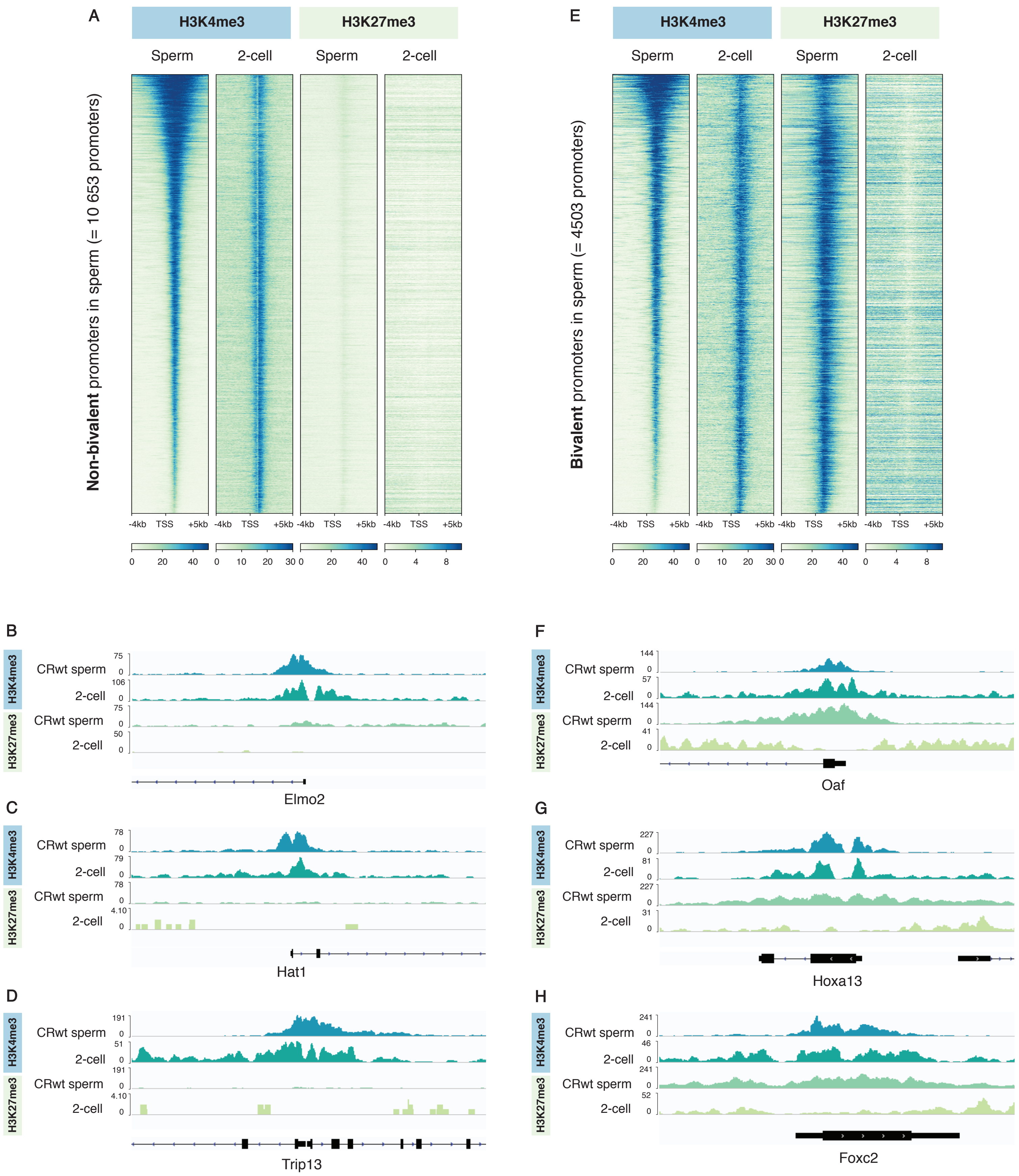
A
H3K4me3
H3K27me3
Sperm
2-cell
Sperm
2-cell
Non-bivalent promoters in sperm (= 10 653 promoters)
-4kb TSS +5kb
0 20 40
0 10 20 30
0 20 40
0 4 8
E
H3K4me3
H3K27me3
Sperm
2-cell
Sperm
2-cell
Bivalent promoters in sperm (= 4503 promoters)
-4kb TSS +5kb
0 20 40
0 10 20 30
0 20 40
0 4 8
B
H3K4me3
H3K27me3
CRwt sperm
2-cell
CRwt sperm
2-cell
Elmo2
C
Hat1
D
Trip13
F
Oaf
G
Hoxa13
H
Foxc2

Figure S8

A

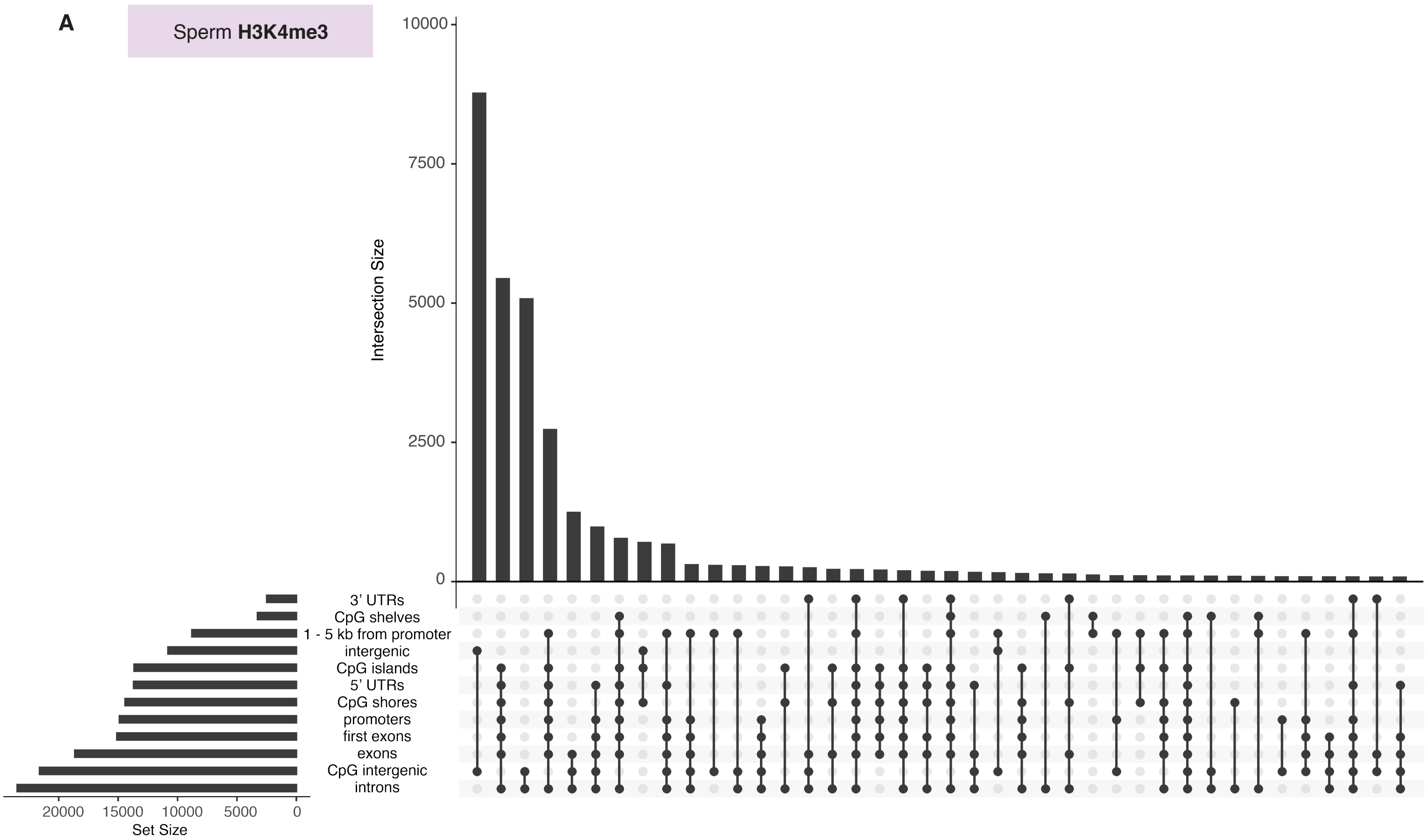
Sperm H3K4me3
10000
7500
5000
2500
0
Intersection Size
3' UTRs
CpG shelves
1 - 5 kb from promoter
intergenic
CpG islands
5' UTRs
CpG shores
promoters
first exons
exons
CpG intergenic
introns
20000 15000 10000 5000 0
Set Size

B

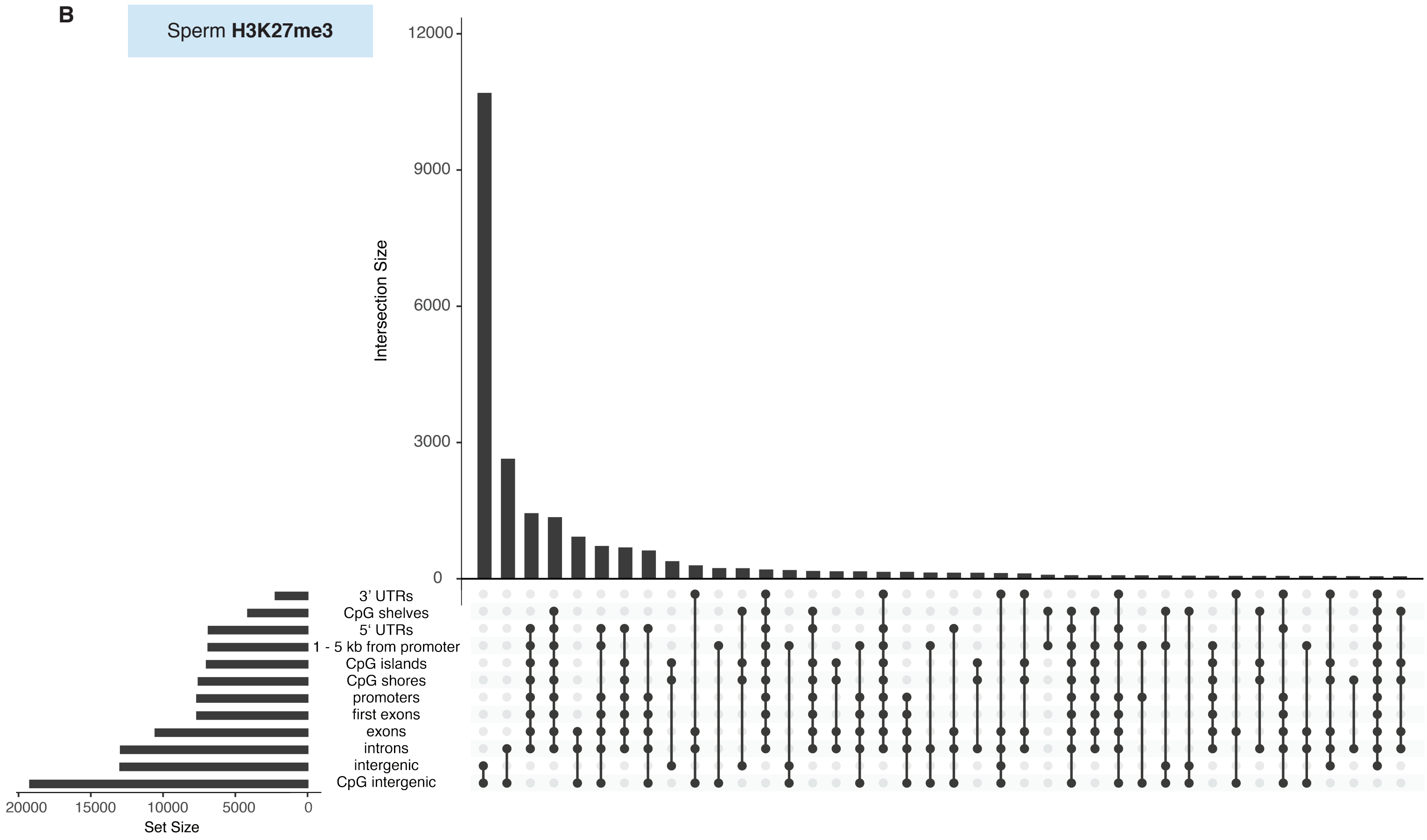
Sperm H3K27me3
12000
9000
6000
3000
0
Intersection Size
3' UTRs
CpG shelves
5' UTRs
1 - 5 kb from promoter
CpG islands
CpG shores
promoters
first exons
exons
introns
intergenic
CpG intergenic
20000 15000 10000 5000 0
Set Size
