# Supplementary material for: Sperm histone H3 lysine 4 trimethylation is altered in a genetic mouse model of transgenerational epigenetic inheritance
Source: Nucleic Acids Res. 2020 Oct 17;48(20):11380–93. doi: 10.1093/nar/gkaa712 (PMC7672453; doi:10.1093/nar/gkaa712)
Supplement: gkaa712_Supplemental_Files [file gkaa712_supplemental_files.zip › NAR_supp_material.docx]

**SUPPLEMENTAL FIGURE LEGENDS**

**Figure S1: Sperm ChIP-Seq for H3K4me3 and H3K27me3 produce reproducible data.**

Pairwise comparisons of log2 read counts for all **(A)**H3K4me3 and **(B**) H3K27me3 replicate samples within 5000 random genome wide tiling windows spanning 2000 bp.

**Figure S2: Normalization and visualization of sperm H3K4me3 and H3K27me3 ChIP-Seq datasets.**

(**A**) Histogram of background read abundance as determined by the number of H3K4me3 ChIP-Seq reads in 2000 bp windows tiled across the genome. An abundance threshold was set at ≥ log2(5) fold over background (red line). Windows below this threshold were filtered out for downstream analysis (see Methods). (**B** - **C**) MA-plots of paired comparisons between CRwt_A and all biological replicates for H3K4me3 positive windows in non-normalized (**B**) and loess-normalized (**C**) data. The log-fold difference in read counts between windows (M values) were plotted against mean read count abundances (A values). (**D**) Histogram of background read abundance as determined by the number of H3K27me3 ChIP-Seq reads in 2000 bp windows tiled across the genome. An abundance threshold was set at ≥ log2(7) fold over background (red line). Windows below this threshold were filtered out for downstream analysis (see Methods). (**E** - **F**) MA-plots of paired comparisons between CRwt_A and all biological replicates for H3K27me3 positive windows in non-normalized (**E**) and loess-normalized (**F**) data. The log-fold difference in read counts between windows (M values) were plotted against mean read count abundances (A values).

**Figure S3: Establishment of background abundance thresholds to identify H3K4me3 and H3K27me3 enrichment at promoters in sperm**.

Distributions of log2 (counts + 8) +/- 1 kb around TSS in CRwt sperm for (**A**) H3K4me3 and (**B**) H3K27me3 datasets. The local minimum was identified on the density plots (red line) on the right and used as the cutoff threshold value to identify promoters enriched for H3K4me3 (purple) and H3K27me3 (blue) in sperm. Counts for each mark are plotted against CpG density.

**Figure S4: Establishment of background abundance thresholds to identify KDM1A, H3K4me2, and H3K4me3 enrichment at promoters in spermatocytes, sperm, and 2-cell embryos, respectively**.

Distributions of log2 (counts + 8) +/- 1 kb around TSS for (**A**) KDM1A spermatocyte (42) and (**B**) H3K4me2 sperm (1) datasets. The local minimum was identified on the density plots (red line) on the right and used as the cutoff threshold value to identify promoters enriched for KDM1A in spermatocytes (blue) and H3K4me2 in sperm (blue) in sperm as visualized on the scatterplot depicting counts according to CpG density for each mark. (**C**) 2-cell H3K4me3 (23) density distribution revealed no bimodal distribution therefore cutoff threshold to identify promoter with enriched H3K4me3 in 2-cell embryos was set empirically (blue dots on H3K4me3 2-cell embryo enrichment vs CpG scatterplot).

**Figure S5: Sperm H3K4me3 and H3K27me3 are enriched at CpG dense loci.**

Upset plots indicating the location of H3K4me3 (**A**) or H3K27me3 (**B**) regions in sperm based on genomic and CpG annotations (annotated with the R/Bioconductor annotatr package) (73). The set size bar graph describes the number of H3K4me3 (**A**) or H3K27me3 (**B**) regions that correspond to a given annotation. Annotations that overlap are represented by connecting nodes. The number of overlapping annotations for H3K4me3 (**A**) or H3K27me3 (**B**) regions is described by the intersection size bar graph. CpG shores are defined as +/- 2 kb from the ends of CpG islands. CpG shelves are defined as another +/- 2 kb from the outer limits of CpG shores. The remaining genomic regions make up the intergenic CpG annotations.

**Figure S6: deH3K4me3 in TG and nonTG sperm are mostly found at non-bivalent loci and H3K27me3, H3K9me2, H3K9me3 are not bound by KDM1A in spermatocytes.**

(**A**) Venn diagram showing the overlap between deH3K4me3 promoters in TG sperm, deH3K4me3 promoters in nonTG sperm, and H3K27me3-enriched promoters in CRwt sperm. (**B** and **C**) Integrative Genome Viewer tracks of the (B) Foxa1 and (C) Hoxd11 promoters marked by H3K27me3 and do not have deH3K4me3 in TG or nonTG sperm. (D - H) Heatmaps at +/- 3 kb of center of regions of KDM1A enrichment in pachytene spermatocytes (= 31 321 regions, see Methods) for KDM1A enrichment in spermatocytes (**D**), and H3K4me3 (**E**), H3K27me3 (**F**), H3K9me2 (**G**), H3K9me3 (**H**) in spermatocytes (KDM1A spermatocyte dataset retrieved from Chen et al. GSE132446) (45).

**Figure S7: H3K27me3 at bivalent chromatin in sperm is absent in the 2-cell embryo.**

(**A**) Heatmaps at - 4kb and + 5 kb around the TSS of non-bivalent promoters in sperm (= 10 653 promoters) for H3K4me3 enrichment in sperm and 2-cell embryos, and H3K27me3 enrichment in sperm and 2-cell embryos. (**B** - **D**) Integrative Genome Viewer tracks of the (**B**) Elmo2, (**C**) Hat1 and (**D**) Trip13 promoters showing H3K4me3 enrichment in sperm and 2-cell embryos but a H3K27me3 depletion in sperm and 2-cell embryos. (**E**) Heatmaps at - 4kb and + 5 kb around the TSS of bivalent promoters in sperm (= 4503 promoters) for H3K27me3 enrichment in sperm and 2-cell embryos, and H3K27me3 enrichment in sperm and 2-cell embryos. (**F** - **H**) Integrative Genome Viewer tracks of the (**F**) Oaf, (**G**) Hoxa13 and (**H**) Foxc2 promoters showing H3K4me3 enrichment in sperm and 2-cell embryos, H3K27me3 enrichment in sperm, and H3K27me3 loss in 2-cell embryos (2-cell embryo H3K4me3 and H3K27me3 datasets retrieved from Liu et al. GSE73952, see Methods) (23).

**Figure S8: Loci of high, medium and low H3K4me3 or H3K27me3 levels in sperm are differentially enriched in genes expressed during different stages of spermatogenesis.**

Gene expression values from various stages of spermatogenesis were retrieved from Gan *et al.* (44). Genes belonging to the top quarter of expression level were used to characterize each spermatogenesis stage (Table S2, see Methods). (**A** and **B**) Hypergeometric tests assess the significance of overlap between each gene set enrichment characterizing stages of spermatogenesis and promoters with high, medium, or low levels of H3K4me3 (A) or H3K27me3 (**B**) in sperm. Dashed line corresponds to a p-value of 0.05.

**Table S1: Sperm H3K4me3 and H3K27me3 ChIP-Sequencing read statistics**

**Table S2: Curated gene lists including the most expressed genes during different spermatogenesis stages based on FPKM values (top quartile)**

**Table S3: Significant gene ontology pathways for high, medium, low levels of H3K4me3 or H3K27me3 enrichment in sperm**
