# Supplementary material for: Sperm histone H3 lysine 4 trimethylation is altered in a genetic mouse model of transgenerational epigenetic inheritance
Source: Nucleic Acids Res. 2020 Oct 17;48(20):11380–93. doi: 10.1093/nar/gkaa712 (PMC7672453; doi:10.1093/nar/gkaa712)
Supplement: gkaa712_Supplemental_Files [file gkaa712_supplemental_files.zip › tableS1_read_stats.pdf]

Sample ID	Factor	Aligned Reads*	% Duplicate	Read Length	% Reads in Blacklist	n regions
CRwt-A	H3K4me3	37040546	28.4	100	10.6	
CRwt-B	H3K4me3	41447857	23.4	100	7.9	
CRwt-C	H3K4me3	37089922	23.8	100	10.5	
nonTG4-A	H3K4me3	38265903	28.5	100	10.4	
nonTG4-B	H3K4me3	33694577	25.2	100	9.0	36,418
nonTG4-C	H3K4me3	40050485	32.3	100	8.0	
TG4-A	H3K4me3	28528809	22.6	100	11.7	
TG4-B	H3K4me3	33318848	27.3	100	8.1	
TG4-C	H3K4me3	31946545	28.5	100	10.9	
C57BL6_A	H3K27me3	96759916	21.5	100	26.0	
C57BL6_B	H3K27me3	87664431	13.8	100	20.2	26,526
nonTG3	H3K27me3	85039866	18.7	100	18.1	
nonTG4	H3K27me3	83743327	24.2	100	22.9	
TG4	H3K27me3	77381993	23.0	100	34.1	
TG3	H3K27me3	81058619	22.2	100	44.5	

* Alignment quality score > 15
